# Novel potential neuroprotective targets for DengZhanXiXin injection in middle cerebral artery occlusion rats recommended by quantitative proteomics and simulated docking

**DOI:** 10.3389/fnins.2025.1499214

**Published:** 2025-07-07

**Authors:** Min Li, Linshuang Wang, Haiting An, Xin Li, Yaojing Chen, Dongfeng Wei, Zhanjun Zhang

**Affiliations:** ^1^State Key Laboratory of Cognitive Neuroscience and Learning and IDG/McGovern Institute for Brain Research, Beijing Normal University, Beijing, China; ^2^BABRI Centre, Beijing Normal University, Beijing, China; ^3^Institute of Basic Research in Clinical Medicine, China Academy of Chinese Medical Sciences, Beijing, China; ^4^Beijing Neurosurgical Institute, Beijing Tiantan Hospital, Beijing, China

**Keywords:** DengZhanXiXin injection, ischemic stroke, MCAO, molecular docking, cell signaling

## Abstract

Stroke, which leads to death and disability in high proportions globally, is one of the most deleterious neurological diseases. Ischemic stroke (IS) is the major cause of disease attack and accounts for ~70% of all incident stroke cases in China. Up to now, only two therapies for IS were officially approved, which are intravenous administration of recombinant tissue-plasminogen activator (rt-PA) and endovascular mechanical thrombectomy to rapidly recanalize the occluded artery, which both recanalize the occluded artery rapidly to reduce disability, but are limited in a fixed time window. In this study, the therapeutic effect of a traditional Chinese medicine, DengZhanXiXin injection (DZXI), was evaluated on middle cerebral artery occlusion (MCAO) rats at the neurobehavioral and pathophysiological levels through neurological tests, neurohistological staining, proteomic assay, and biological information analysis. We found that DZXI significantly ameliorated the neurological deficit, prevented infarct volume evolution, and protected cortical neural cells from death in ischemia penumbra on MCAO rats. Furthermore, corresponding therapeutic molecular targets were investigated through proteomic analysis of ischemic hemispheres of MCAO rats. One hundred ninety-one differentially expressed proteins involved in response to metal ions, neurofilament bundle assembly, and modulation of chemical synaptic transmission were identified between the MCAO model and DZXI groups after 7 days. DZXI influenced the expression levels of proteins in 13 specific biological functions, with cell signaling and chemical synaptic transmission-associated proteins being most affected. Subsequent molecular docking analysis predicted binding potential between key target proteins and DZXI compounds. The results suggested that DZXI ameliorates neurological deficits by potentially affecting cellular signaling and chemical synaptic transmission physiological processes.

## Introduction

As one of the most damaging neurological diseases, stroke is the second highest cause of death and a leading cause of disability globally (GBDS Collaborators, 2021). China has the highest number of prevalent cases of stroke in the world, affecting ~3 million people (Wang W. et al., 2017). Among them, ischemic stroke (IS) accounts for ~70% of all incident stroke cases based on a Chinese nationwide community study (Wu et al., 2019). It is caused by a sudden disruption of blood flow due to arterial occlusion, resulting in the sudden onset of a focal neurological deficit. The clinical features of IS are greatly related to location, volume, and number of presented infarcts, such as right hemiparesis with aphasia due to occlusion of the left middle cerebral artery (Campbell and Khatri, 2020). Ischemic stroke has a serious effect on individuals, their caregivers, and society. To date, the major approved therapy for IS patients involves rapid recanalization of the occluded artery through intravenous thrombolysis and endovascular mechanical thrombectomy in cases with large-vessel occlusions, which reduces disability but is time-critical (Powers et al., 2019). There is still the key challenge of extending therapeutic advantages to broader IS patients. Therefore, minimizing usage constraints drives the exploration of more innovative therapeutic targets for drug development.

Most IS are triggered by cerebral embolism, which may come from atherosclerotic plaque in the aortic arch, carotid artery, or the heart. Furthermore, intracranial atherosclerosis with *in situ* thrombosis is also an important mechanism of IS. Occlusion of a cerebral artery reduces cerebral blood flow and initiates a cascade of events, including depleted ATP stores, irreversible failure of energy metabolism, excitotoxicity and calcium overload, mitochondrial alterations, reactive oxygen species (ROS) production, protein misfolding, and inflammatory response leading to neural cell loss (George and Steinberg, 2015; Moskowitz et al., 2010). Ischemia results in a deficiency of glucose and oxygen, causing the inability of neurons to maintain normal transmembrane ionic gradients (Campbell et al., 2019). Furthermore, anoxic depolarization at presynaptic terminals leads to excessive glutamate release (Obrenovitch et al., 1993) and further results in the intracellular influx of calcium, triggering cell death pathways (Lipton, 1999), which is termed excitotoxicity. The intracellular increase in calcium also triggers mitochondrial dysfunction, free radical overproduction, and activation of proteases and phospholipases, which are neurotoxic (George and Steinberg, 2015; Szydlowska and Tymianski, 2010). Numerous therapeutic approaches have focused on blocking pathways associated with excitotoxicity to improve stroke recovery, although they often have efficacy in animal models (Yenari et al., 2001; Namura et al., 2013) and translation of them into the clinic remains challenging. Furthermore, the inflammatory response is another principal systemic example that both helps propagate ischemic injury and promotes recovery. Inflammation initially contributes to cellular injury by releasing cytokines and detrimental radicals (Huang et al., 2006), but eventually helps to remove damaged tissue (Stephan et al., 2012), enabling synaptic remodeling (Lalancette-Hébert et al., 2007; Wang et al., 2013). The neuroprotective efficacy of modulating these pathological processes in ischemia remains to be fully elucidated and warrants further systematic investigation.

*Erigeron breviscapus* (Vant.) Hand.-Mazz. is a traditional Chinese medicinal plant mainly grown in southwest China and has a long medicinal history in Chinese medicine (Chai et al., 2013). There are numerous preparations that have been extensively used in clinics in China to treat ischemic cardio-cerebral vascular diseases for a long time (Ding and Li, 2009). The DengZhanXiXin injection (DZXI) is a phenolic acid extract from the herb *Erigeron breviscapus*, which has been officially listed in the Chinese Pharmacopeia since 2005 (Commission, 2005) and approved by the China Food and Drug Administration with its approval number Z53021620/Z53021569 (Wang J. et al., 2017). The main active compounds of DZXI include scutellarin, 3,4-O-dicaffeoylquinic acid, 3,5-O-dicaffeoylquinic acid, erigoster B, 4,5-O-dicaffeoylquinic acid, and erigeroster (Lin, 2020). DZXI has been clinically applied to treat IS, coronary artery disease, stenocardia, and other cardio-cerebral vascular diseases (Li et al., 2017; Wang et al., 2015). The mechanism of DZXI, as listed in its instructions, is that DZXI can activate blood circulation to dissipate blood stasis and relieve pain. Previous research showed that DZXI suppresses platelet aggregation and reduces blood viscosity to improve blood supply for ischemic neural cells (Lin et al., 2003). Furthermore, it can also upregulate neurotrophic factors synthesis and release in hypoxia/reoxygenation astrocytes (Chai et al., 2013) and exhibit strong antioxidation by inhibiting protein kinase C (PKC; Li et al., 2017). These findings suggest that DZXI may represent a promising therapeutic candidate for rescuing acute ischemia injury to ameliorate focal clinical deficit in IS patients.

In the present study, we investigated the ameliorative effect of DZXI intervention and explored molecular therapeutic targets in rats with middle cerebral artery occlusion (MCAO), a model with reliable and well-reproducible infarcts, highly mimicking human IS in the majority of studies that investigated pathophysiological processes and neuroprotective agents for IS (Fluri et al., 2015). We examined behavioral tests, neurohistological staining, proteomic assay, and biological information analysis in the rat stroke model with DZXI intervention. This study explores potential target proteins for DZXI, providing insights into its pharmacological mechanism, which helps develop more effective IS drugs and benefits more IS patients.

## Materials and methods

### Drugs

DengZhanXiXin injection (specifications: 5.32 mg scutellarin, 2.26 mg 3,4-O-dicaffeoylquinic acid, 1.10 mg 3,5-O-dicaffeoylquinic acid, 1.79 mg erigoster B, 2.70 mg 4,5-O-dicaffeoylquinic acid, and 11.26 mg erigeroster, 10 ml/ampoule, Lot No. 20180137) was provided by Yunnan Biovalley Pharmaceutical Co., Ltd (Kunming, China).

### Animals and grouping

The animal experiments were conducted under the direction of NIH Guidelines for the Care and Use of Laboratory Animals (NIH Publications No. 80–23, revised 1996). The procedures were approved by the Animal Care and Use Committees of Beijing Normal University, China. Adult male Sprague-Dawley rats (270 ± 10 g) were purchased from Beijing Vital River Laboratory Animal Technology Co., Ltd. The animals were allowed to acclimate for 7 days before the experiments. Rats were randomly divided into a sham-operated (sham) group, MCAO model group, and DZXI-treated (DZXI) group (*n* = 10 rats each group). Animals were housed in cages in a controlled environment (22–25°C, 50% humidity, and a 12-h light/dark cycle) with free access to standard laboratory chow and distilled water. All efforts were made to minimize animal suffering and the number of animals used.

### Middle cerebral artery occlusion surgery

All rats were fasted overnight before surgery but allowed free access to water. The rats were anesthetized with 3.5% chloral hydrate (350 mg/kg body weight, i.p.), and MCAO surgery was performed. Focal cerebral ischemia was induced using the filament model as described in previous research (Mhairi Macrae, 1992). Briefly, the right common carotid artery (CCA) and external and internal carotid arteries (ECA and ICA) were exposed through a midline cut. Temporarily clamp the CCA with an arterial clip, ligate the proximal bifurcation of the ECA and the proximal end of the CCA, then clamp the ICA with an arterial clip, and make a small incision between the CCA ligation point and the clamping point. A nylon monofilament coated at the tip with 5 mm of silicone (diameter 0.36 ± 0.02 mm) was inserted into the lumen of the right ICA through the CCA. Keep inserting until encountering slight resistance, then the tip reaches the entrance to the middle cerebral artery (MCA). The length of the inserted filament was around 18–22 mm from the carotid bifurcation point. Then, the CCA was ligated to fix the filament and prevent bleeding. After a 90-min ischemic period, the occluding filament was gently withdrawn to restore the blood flow for reperfusion injury, followed by surgical site suturing. The rectal temperature was maintained at 37 ± 0.5°C with a thermostatically controlled heating blanket throughout the surgical procedure. In the sham group, animals were subjected to the same procedure, except for the insertion of the nylon monofilament.

### Drug treatment

DZXI (0.18 mL/100 g) was intravenously injected into rats in the DZXI group. The concentrations of main active compounds (in 10 ml/ampoule): 5.32 mg scutellarin, 2.26 mg 3,4-O-dicaffeoylquinic acid, 1.10 mg 3,5-Odicaffeoylquinic acid, 1.79 mg erigoster B, 2.70 mg 4,5-O-dicaffeoylquinic acid, and 11.26 mg erigeroster. The first drug treatment was conducted immediately after the MCAO surgery, and subsequent treatments were continually performed at an interval of 12 h, i.e., twice a day. The drug treatments lasted for 7 days. The dosage and treatment course referred to general usage for humans and the equivalent dose ratio based on body surface area (An et al., 2021). The rats in the sham group and model group were treated with the same volume of saline in the same way.

### Neurobehavioral assessment

Modified neurological severity score (mNSS) test was conducted by raters who were blind to animal grouping to measure neurological function at 6 h, 24 h, and 7 days after MCAO and DZXI treatment. The mNSS test is the standard and globally accepted method to evaluate the severity of post-stroke injury and recovery. The mNSS test comprised motor, sensory, reflex, and balance tests. The total score of mNSS ranges from 0 to 18, and a higher test score indicates a more severe neurological deficit (Chen et al., 2001).

### 2,3,5-triphenyltetrazolium chloride (TTC) staining

Infarction caused by MCAO was confirmed by TTC staining to assess infarction size (Bederson et al., 1986). The rats were sacrificed 7 days after MCAO and drug treatment. The rat brains were rapidly removed and frozen at −20°C for 30 mi, after which 2 mm thick coronal sections of the rat brains were cut. The brain sections were incubated with 1% TTC in the dark for 20 min at 37°C. After TTC staining, sections were fixed in 4% polyformaldehyde buffered solution for 20 min. The cerebral infarct area was outlined in white in MCAO rats.

### Hematoxylin-eosin staining

Pathological alterations in the morphology of neural cells around the focal ischemic area, also referred to as the ischemic penumbra, were evaluated by hematoxylin-eosin (HE) staining. After the rats were anesthetized with 3.5% chloral hydrate, they were perfused with warm saline via the left ventricle, and their brains were fixed with 2% glutaraldehyde and 4% paraformaldehyde (PFA). Then the whole brain was embedded in paraffin and serially coronally sectioned. The sections were dewaxed with xylene, dehydrated with a gradient of alcohol solutions, and washed with running water. After this, the brain sections were stained with haematoxylin and differentiated with 0.5% hydrochloric acid alcoholic solution, then washed with running water. Then, the sections were returned to a blue color by incubating them with a saturated lithium carbonate solution for 1 min and stained with a 0.1–0.5% eosin solution for 10 min. The sections were examined using a light microscope and then photographed. Moreover, the numbers of dead neural cells in sections of rats in each group were counted using ImageJ software. The ratio was calculated as the number of dead neural cells divided by the number of total neural cells in images. Notably, histological staining was accomplished by raters who were blind to the animal grouping situation.

### Proteomic analysis

In the sham, model, and DZXI groups, rats were sacrificed 7 days after surgery, and ischemic hemispheres (four replicates in each group) containing the ischemic core were harvested. The samples were digested with trypsin and labeled with TMT (tandem mass tags, Thermo). An equal amount of each labeled sample was mixed, chromatographically separated, and finally subjected to an LC-MS/MS analysis. The peptide mass maps were analyzed using Proteome Discoverer^TM^ 2.2 software (Thermo) and the UniProt database. The significantly differential proteins between the two groups were identified with a fold change (FC) >1.2 and *p*-value (calculated by *t*-test) < 0.05. These differentially expressed proteins were entered as foci into the subsequent biological information analysis.

### Biological information analysis

To interpret experimental information and discover potentially valuable proteins, the proteomic data were analyzed using biological information from the Gene Ontology (GO) and STRING databases. GO enrichment analysis of 191 differentially expressed proteins was performed on the Metascape platform to classify these proteins according to their biological process (Zhou et al., 2019). GO enrichment analysis refers to the distribution of experimental data, which was compared with the distribution of the overall protein, confirming experimentally identified proteins were significantly enriched in which categories. Subsequently, to further understand the biological context of the differentially expressed proteins, protein interaction analysis was carried out using the free website search tool STRING11.5. The STRING database (https://string-db.org) systematically collects and integrates known and predicted protein-protein interaction data, both physical interactions and functional associations, for many organisms (Szklarczyk et al., 2023). The gene symbol list of 191 differentially expressed proteins was input into the STRING database to identify known and predicted protein-protein interaction networks.

### Molecular docking simulation

The potential binding capacity between the main compounds of DZXI and key protein targets was analyzed by molecular docking using the Autodock Vina and AutoDock (Trott and Olson, 2010). The 3D structures of the main compounds were obtained from the TCMSP database, whereas the key protein targets' 3D structures were obtained from the RCSB Protein Data Bank (PDB) and AlphaFold. The figures of the active binding sites between chemical compounds and key proteins were generated with the PyMOL software (Burley et al., 2017).

### Statistical analysis

Neurobehavioral data were analyzed using two-way ANOVA, followed by Turkey's HSD test to complete *post-hoc* multiple comparisons. Statistical figure was drawn in Prism 8.0 with data expressed as mean ± SD. The difference in infarction ratio and dead cell ratio between groups was analyzed using one-way ANOVA and Turkey's HSD test, then plotted in R. Differential analysis for proteomic data was calculated by Student's *t*-test with FDR correction in R. The visualization of protein expression data was conducted in R. The criterion for statistical significance was set as *p* < 0.05.

## Results

### DZXI treatment prevented infarct evolution and ameliorated motor impairment in MCAO rats

**Figure 1:**
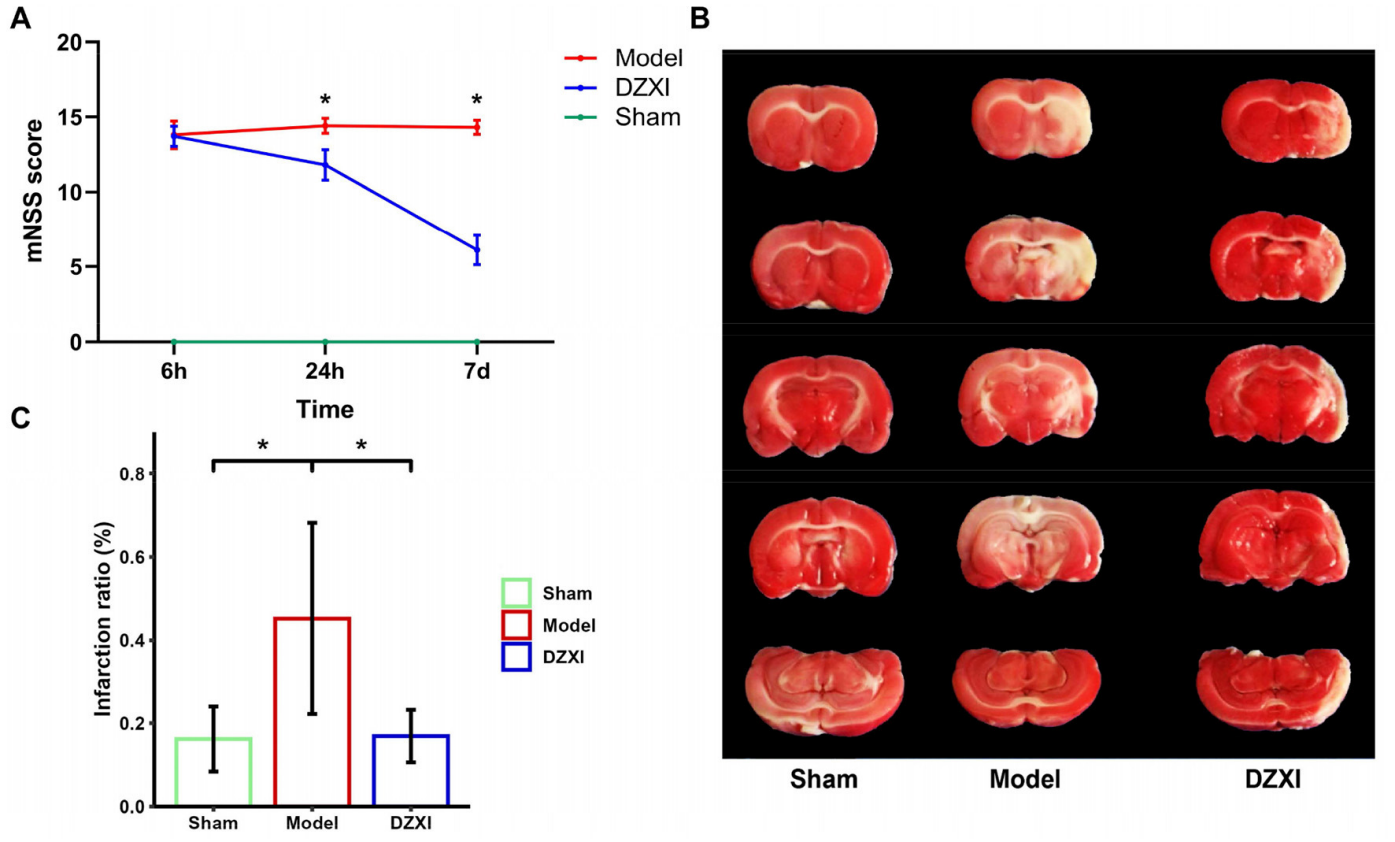
**(A)** mNSS score of the rats in each MCAO and DZXI treatment group. Red, blue, and green lines represent the mean scores of MCAO, DZXI, and sham groups, respectively (*n* = 10 in each group). Error bars indicate SD. The time and group effects on mNSS score were analyzed by two-way ANOVA followed by Turkey's HSD test, *P < 0.05, DZXI vs. model. **(B)** TTC staining of rat brain serial coronal slices in sham, MCAO, and MCAO with DZXI treatment groups. TTC staining showed red healthy zones and pale infarcted regions. **(C)** The infarction ratio in different groups was plotted (*n* = 5 in each group). Error bars indicate SD. The group difference was analyzed with one-way ANOVA followed by Turkey's HSD test, *P < 0.05.

To evaluate the potential neuroprotection activity of DZXI in focal cerebral ischemia, rats were subjected to MCAO surgery. The severity of neurological deficits, such as hemiparesis and motor coordination, is a crucial issue for the evaluation of stroke consequences. After 6 h, 24 h, and 7 days of treatment, the post-stroke neurological deficits of the experimental rats in each group were scored using the mNSS scoring criterion. The results of mNSS showed that DZXI intervention could significantly improve the neurological function recovery of ischemia-reperfusion injury rats compared with MCAO rats (Figure 1A). Besides, MCAO was validated by TTC staining, where infarction in the right caudate putamen and temporal lobe cortex could be observed 7 days post-MCAO. Compared with the typical image of the sham group, the infarct size of the MCAO group was significantly increased after MCAO injury. When treated with DZXI, TTC staining images showed that the infarct volume in the DZXI group significantly reduced compared with the MCAO group (Figures 1B, C).

### DZXI treatment exhibited neuroprotection in the ischemic penumbra in MCAO rats

**Figure 2:**
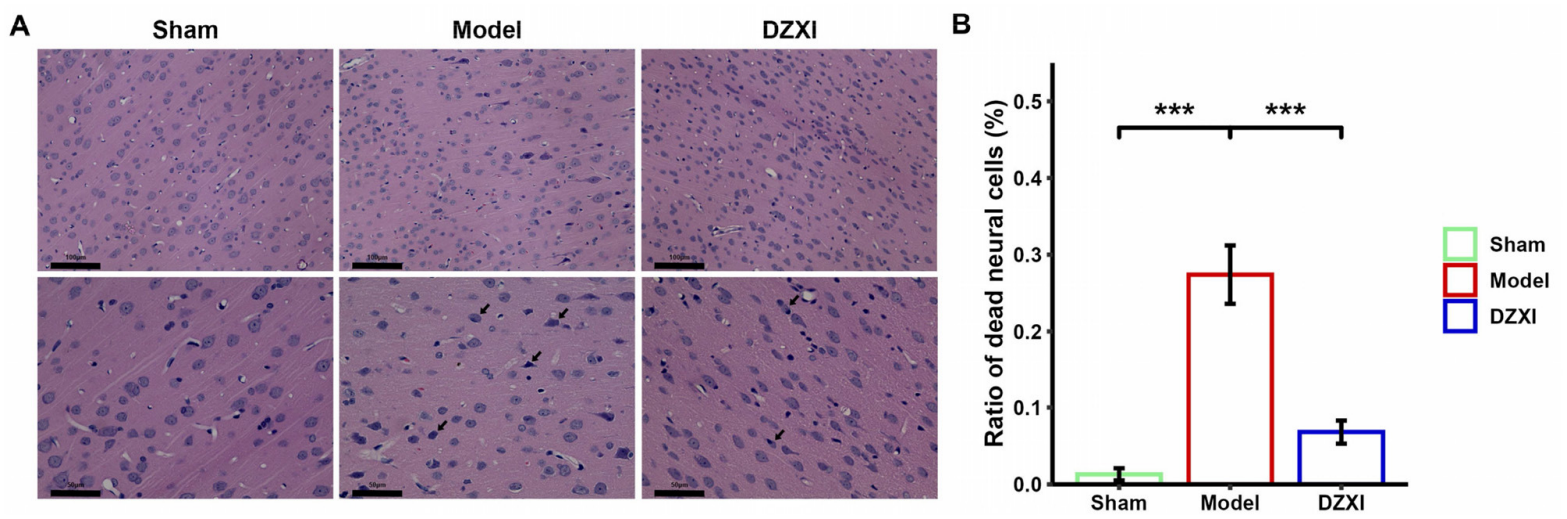
**(A)** The morphological change of cortical penumbra neural cells in different groups was examined by HE staining. The magnification of the upper images is smaller for observing the overall arrangement of cortical neural cells, while the magnification of the lower images is larger, allowing for a detailed observation of the structure and morphology of neural cells. The black arrows in the image indicate dead cells with swollen morphology, blurred structure, or nuclear condensation. The scale bar is displayed in the lower left corner of the image. **(B)** The ratios of dead neural cells vs. total neural cells in different groups were plotted (*n* = 5 in each group). Error bars indicate SD. The group difference was analyzed with one-way ANOVA followed by the Turkey's HSD test, ***P < 0.001.

Besides, we examined the morphology of cortical neural cells in ischemic penumbra through HE staining. In the brain tissue of sham group, the morphology of neural cells was normal, and no pathological degeneration or necrosis of nerve cells was observed (Figure 2A left). In contrast, in the MCAO group, the penumbra tissue cells were arranged irregularly and appeared to have more cellular swelling and necrosis with a certain degree of fuzzy neural structure and deep nuclear staining (Figure 2A middle). Moreover, compared with the sham group, the number of dead neural cells stained by HE staining increased in the cortex of MCAO rats (Figure 2B). After DZXI treatment, the cortical penumbra neural cells exhibited restored intact morphological features, including more orderly arrangement and clearer cell structures (Figure 2A, right). Furthermore, the number of dead neural cells in the cortical penumbra after DZXI treatment significantly declined compared with the MCAO group (Figure 2B). These results illustrated that the DZXI treatment could protect neural cells from ischemic injury, possibly associated with its ameliorative effect on IS.

### Identification of the differentially expressed proteins explored the DZXI neuroprotective effect at the protein level

**Figure 3:**
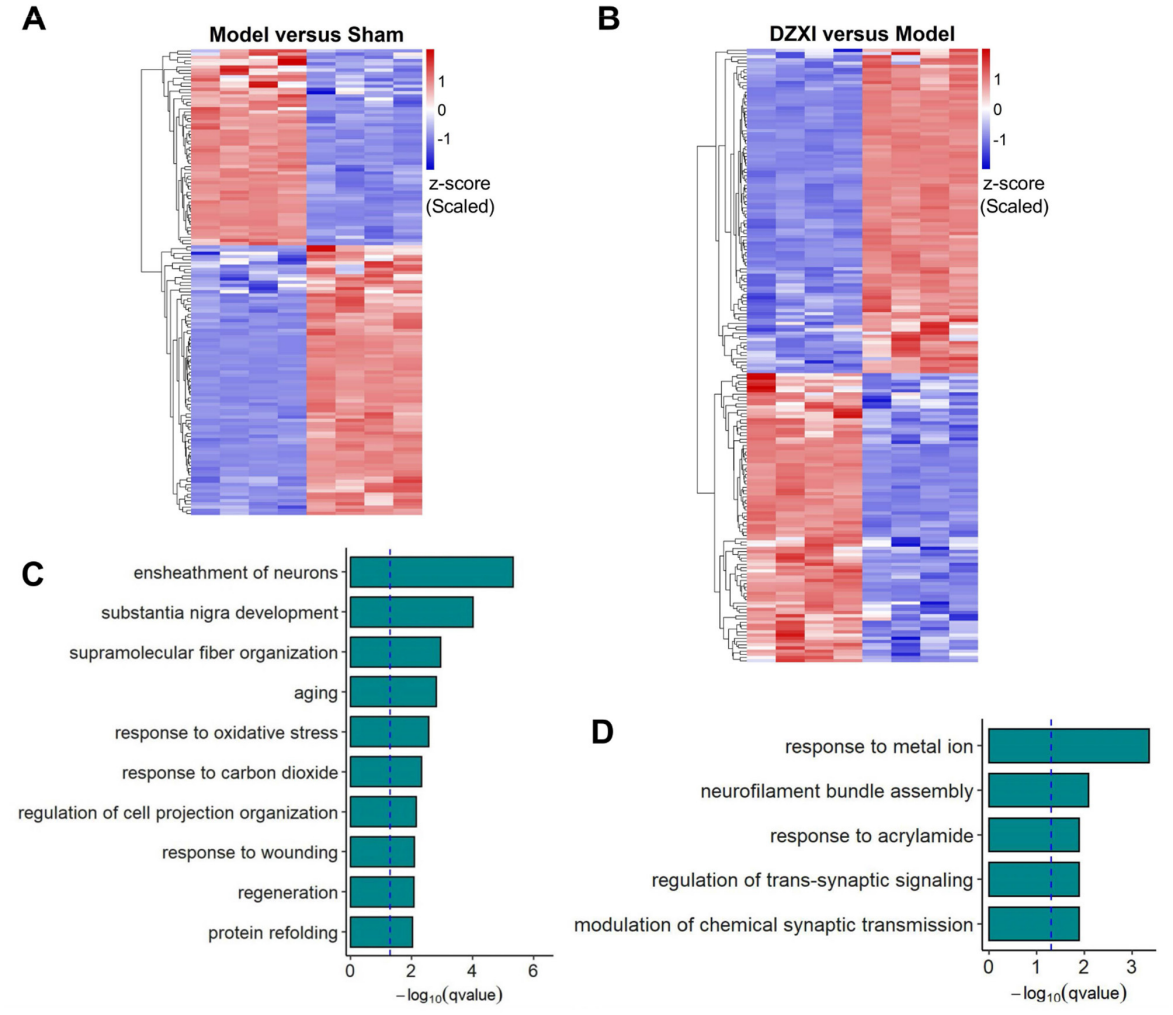
**(A)** The DEPs between the sham group and MCAO model group identified in proteomic assays were clustered according to their expression levels. **(B)** The DEPs between the MCAO model group and DZXI group identified in proteomic assays were clustered according to their expression levels. The relative TMT intensities of each protein (rows) in each group (columns) were indicated on a colored scale in both **(A, B)**, where red represents a high expression level and blue represents a low expression level. **(C)**. The top 10 biological processes enriched in the DEPs between the sham group and the MCAO model group were enriched. **(D)** The top 5 biological processes in which the DEPs between the model group and DZXI group were enriched. Both results in **(C, D)** were acquired with GO analysis.

Through the proteomic detection and analysis, we identified 145 differentially expressed proteins (DEPs) between the sham and MCAO model groups and 191 DEPs between the model and DZXI groups after 7-day treatment (Supplementary Table S1). The result of clustering analysis for these proteins is depicted in Figures 3A, B. The GO analysis of DEPs between the sham group and MCAO model groups showed that ensheathment of neurons, response to oxidative stress, response to carbon dioxide and response to wounding were significantly enriched biological processes items (Figure 3C), whereas response to metal ion, neurofilament bundle assembly and modulation of chemical synaptic transmission were most enriched items of DEPs between the model and DZXI groups (Figure 3D). The GO enrichment analysis illustrated that the protein changes caused by acute ischemic injury primarily reflected the large number of cell deaths that leads to significant reduction in protein components involved in neuronal myelin formation, as well as the response to ischemia and hypoxia injury (such as oxidative stress, response to carbon dioxide, and response to injury). Moreover, the protein changes due to DZXI treatment mainly concentrated on proteins that respond to metal ions (such as calcium dependent proteins), proteins involved in neurofilament bundle assembly, and proteins related to trans-synaptic signaling and chemical synaptic transmission (such as chemical neurotransmitter receptors), suggesting that these protein clusters could be potentially influenced by DZXI treatment.

### DZXI treatment modulated several specific protein networks in MCAO rats

Furthermore, we selected and categorized 39 principal proteins among DEPs between the MCAO model and DZXI groups after 7-day treatment based on their specific functions. The detailed information of these proteins, including accession number, protein name, gene symbol, MW, pI, protein score, and fold change, is summarized in Table 1, and their specific functions are displayed in Table 2. The protein expression levels of 23 proteins were decreased, and 16 proteins were increased after DZXI treatment. These proteins were divided into 13 categories according to their specific functions, such as anti-inflammatory, calcium-dependent phospholipid binding, chemical synaptic transmission, cell signaling, energy metabolism, and antioxidation. Among these proteins, eight cell signaling-associated proteins and six chemical synaptic transmission-associated proteins were impacted; the other 25 proteins are involved in anti-inflammatory, calcium-dependent phospholipid binding, energy metabolism, and antioxidation. Therefore, cell signaling and chemical synaptic transmission were the most affected physiological processes during DZXI treatment. As shown in Supplementary Table S2, the molecular function and biological process categories of these 39 proteins were determined based on biological function.

**Table 1:** The identification results of 39 differentially expressed protein spots between the DZXI group and the model group using LC-MS/MS analysis.

**Accession no**.	**Protein name**	**Gene symbol**	**MW (kDa)**	**pI**	**Protein score**	**Fold change (DZXI/Model)**	***P*-value**
P07150	Annexin A1	Anxa1	38.8	7.34	17.42	0.79	<0.001
Q07936	Annexin A2	Anxa2	38.7	7.69	51.81	0.81	<0.001
Q66HH8	Annexin	Anxa5	35.8	5.05	108.61	0.73	<0.05
F1M0Z3	Copine 4	Cpne4	63.3	6.33	85.22	1.37	<0.001
D4ACG7	Copine 6	Cpne6	61.7	5.59	171.19	1.36	<0.001
H1UBN0	Copine-7	Cpne7	61.9	5.40	66.59	1.66	<0.001
G3V6M3	Synaptotagmin II	Syt2	47.2	7.99	253.54	0.67	<0.001
B6DYQ2	Glutathione S-transferase	Gstm2	25.7	7.39	83.06	0.72	<0.001
Q63639	Retinal dehydrogenase 2	Aldh1a2	56.6	5.74	24.94	0.79	<0.005
Q6AY99	Aldo-keto reductase family 1 member B10	Akr1b10	36.0	8.32	51.02	1.20	<0.001
Q09426	2-hydroxyacylsphingosine 1-beta-galactosyltransferase	Ugt8	61.1	9.19	9.89	0.79	<0.05
I7EFB0	Myelin basic protein	Mbp	22.9	10.27	500.34	0.79	<0.001
P60203	Myelin proteolipid protein	Plp1	30.1	8.35	450.37	0.68	<0.001
P19527	Neurofilament light polypeptide	Nefl	61.3	4.65	440.36	0.66	<0.001
P12839	Neurofilament medium polypeptide	Nefm	95.7	4.79	592.94	0.70	<0.001
F1LRZ7	Neurofilament heavy polypeptide	Nefh	114.3	5.81	380.86	0.58	<0.001
B0BNN3	Carbonic anhydrase 1	Car1	28.3	7.42	46.05	0.79	<0.001
P27139	Carbonic anhydrase 2	Car2	29.1	7.40	215.67	0.79	<0.001
P14141	Carbonic anhydrase 3	Car3	29.4	7.37	6.03	0.38	<0.001
M0R9A7	Glutamate receptor 1	Gria1	90.5	8.43	118.4	1.23	<0.001
P31421	Metabotropic glutamate receptor 2	Grm2	95.7	7.80	80.04	1.21	<0.001
Q5BJU5	Protein cornichon homolog 2	Cnih2	18.9	7.25	3.54	1.25	<0.001
D4A4M0	Shisa family member 6	Shisa6	58.1	9.44	5.42	1.53	<0.01
P18508	Gamma-aminobutyric acid receptor subunit gamma-2	Gabrg2	54.0	8.47	42.68	1.23	<0.001
P15431	Gamma-aminobutyric acid receptor subunit beta-1	Gabrb1	54.0	8.76	49.92	1.30	<0.005
G3V874	Erythrocyte membrane protein band 4.1-like 3	Epb41l3	107	5.24	520.06	0.76	<0.001
A0A0G2JWK7	Transgelin	Tagln	23.3	8.66	51.51	0.63	<0.001
B0BMS8	Myl9 protein	Myl9	19.8	4.92	66.17	0.81	<0.001
Q5UAJ6	Cytochrome c oxidase subunit 2	mt-Co2	25.9	4.73	60.46	1.83	<0.001
A0A0H2UHF8	Alpha-1-acid glycoprotein	Orm1	26.7	6.54	9.18	1.22	<0.001
D4A469	Sestrin 3	Sesn3	56.9	6.25	3.50	1.27	<0.001
Q01066	Calcium/calmodulin-dependent3′5′-cyclic nucleotide phosphodiesterase 1B	Pde1b	61.2	5.72	106.5	0.77	<0.001
Q63421	Calcium/calmodulin-dependent3′5′-cyclic nucleotide phosphodiesterase 1C	Pde1c	86.6	8.9	10.37	0.68	<0.001
Q9QYJ6	cAMP and cAMP-inhibited cGMP3′5′-cyclic phosphodiesterase 10A	Pde10a	90.1	6.55	38.35	0.57	<0.001
P63319	Protein kinase C gamma type	Prkcg	78.3	7.46	300.70	1.26	<0.001
A0A140UHX4	Protein kinase AMP-activated non-catalytic subunit gamma 2	Prkag2	62.9	9.42	19.39	1.20	<0.001
P49620	Diacylglycerol kinase gamma	Dgkg	88.5	6.95	52.87	1.32	<0.001
P38406	Adenylate cyclase 5	Adcy5	139	7.06	70.41	0.73	<0.001
P38406	Guanine nucleotide-binding protein G(olf) subunit alpha	Gnal	44.2	6.65	76.45	0.47	<0.001

MW represented the theoretical molecular weight of the proteins. pI represents the theoretical isoelectric point of the proteins. The fold change was represented by the ratio of the TMT intensity of the DZXI group to the value of the model group.

**Table 2:** The specific function and subcellular location of 39 differentially expressed proteins regulated by 7d-treatment of DZXI in the infarcted hemispheres of MCAO rats.

**NO**.	**Gene symbol**	**Specific function**	**Subcellular location**
**Anti-inflammatory proteins**			
1	ANXA1	ANXA1 undergoes Ca^2+^-dependent binding to phospholipids, regulated by glucocorticoids, and has potent anti-inflammatory and pro-resolving properties (Wallner et al., 1986; Purvis et al., 2019).	Nucleus, Cytoplasm, Plasma membrane, Secreted in the extracellular space
2	ANXA2	ANXA2 is a Ca^2+^ regulated phospholipid binding protein involved in cell cycle regulation, cell division, proliferation, cell survival, neo-angiogenesis, and anti-inflammatory response (Sharma, 2019; Dallacasagrande and Hajjar, 2020).	Secreted in the extracellular space, the Melanosome
3	ANXA5	ANXA5 is a single-chain protein known for binding to phosphatidylserine with high affinity in a Ca^2+^-dependent manner. ANXA5 has shown anti-inflammatory, anti-apoptotic, and anticoagulant properties via binding to phosphatidylserine expressed in stressed and dying cells and shielding these cells from inflammatory cell contact (Boersma et al., 2005; van Genderen et al., 2008).	Cytoplasm
**Calcium-dependent phospholipid-binding proteins**			
4	CPNE4	CPNE4 is a Ca^2+^-dependent phospholipid-binding protein that may play a role in membrane trafficking, mitogenesis, and development. CPNE4 is one of the genes downregulated most significantly following mild ischemic exposure in cortical neurons and may participate in cell death or signal transduction (Prasad et al., 2012; Lee et al., 2015).	Plasma membrane
5	CPNE6	CPNE6 is thought to be associated with long-term potentiation (LTP) and spine structural plasticity necessary for learning and memory. Moreover, up-regulated CPNE6 is also closely related to the maturation of axons (Yamatani et al., 2010; Reinhard et al., 2016).	Cytoplasm, Plasma membrane, Axon, Dendrite
6	CPNE7	CPNE7 is capable of Ca^2+^-dependent translocation to the plasma membrane in response to the rise in intracellular calcium. The rapid translocation response of CPNE7 suggests that this protein may play an important role in Ca^2+^-dependent intracellular signaling (Perestenko et al., 2010).	Nucleus, Cytoplasm, Plasma membrane
**Integral synaptic vesicle membrane protein**			
7	SYT2	SYT2 is essential for regulating Ca^2+^-mediated exocytosis. SYT2 is the main isoform expressed at the presynaptic neuromuscular junction and functions as a calcium sensor for neurotransmission (Donkervoort et al., 2020).	Synaptic vesicle membrane
**Detoxification enzymes (Reactive oxygen species (ROS) and xenobiotics metabolism)**			
8	GSTM2	GSTM2 belongs to a large gene family encoding glutathione S-transferases, which catalyze the conjugation of electrophilic compounds to glutathione, thus playing a prominent role in cellular resistance against oxidative stress (McBride et al., 2003).	Cytoplasm
9	ALDH1A2	ALDH1A2 is an enzyme required to convert retinol to retinoic acid, a hormone with diverse functions in the CNS, including neurogenesis and cell survival (Kelly et al., 2016).	Cytoplasm
10	AKR1B10	AKR1B10 is a typical nuclear factor, erythroid 2 (NF-E2)-related factor 2 (Nrf2) target gene. Nrf2 is an oxidative stress-responsive transcription factor for antioxidant genes through binding to its recognition DNA element, the antioxidant responsive element (ARE; Mimura et al., 2019).	Cytoplasm, Secreted in the extracellular space, Lysosome
**Structural constituents of the myelin sheath**			
11	UGT8	Uridine diphosphate (UDP) glycosyltransferases (UGTs) represent a superfamily of enzymes that catalyze the transfer of nucleotide sugars to a large number of exogenous and endogenous compounds to facilitate their elimination from target cells. UGT8 family contains only a single member to date, which encodes a key enzyme in the biosynthesis of glycosphingolipids, cerebrosides, and sulfatides, essential constituents of myelin membranes of the central and peripheral nervous systems (Bosio et al., 1996; Iida et al., 2002).	Cell membrane, Endoplasmic reticulum
12	MBP	MBP is an oligodendrocyte protein and the second most abundant protein in central nervous system myelin, a key structural component of the multi-layered myelin sheath covering nerve fibers (Boggs, 2006).	Myelin membrane
13	PLP1	PLP1 is a highly conserved four-transmembrane-domain oligodendrocyte protein. Excess or abnormal PLP1 will trigger cellular stress responses and oligodendrocyte death. Suppressing expression of PLP1 could increase myelination, restore oligodendrocyte numbers, and nerve conduction velocity (Elitt et al., 2020).	Cell membrane, Myelin membrane
**Neurofilament structural components**			
14	NEFL/M/H	Neurofilament is a highly specific structural protein and a major component of neurons. It consists predominantly of four subunits: neurofilament light, medium, heavy chains, and alpha-internexin. Studies demonstrate that neurofilaments are obligate heteropolymers required for proper radial growth of axons (Iłżecki et al., 2017).	Cytoplasm, Axon
**Carbonic anhydrases**			
15	CAR1	CAR1 is the most abundant non-hemoglobin protein in human erythrocytes. Its physiological function is unclear other than as a backup for CAR2. It might be essential for survival without CAR2 (Sly and Hu, 1995).	Cytoplasm
16	CAR2	CAR2 is a highly active isozyme with a maximum turnover rate for CO_2_ hydration and has the widest distribution. CAR2 accumulates on oligodendrocyte processes associated with myelinated axons, and it is thought that CAR2 may be involved in myelin formation in the CNS (Kida et al., 2006).	Cell membrane, Cytoplasm
17	CAR3	CAR3 catalyzes the reversible reaction between water and CO_2_, thus generating bicarbonate and hydrogen ions to maintain pH homeostasis. CAR3 is rapidly glutathionylated *in vivo* and *in vitro* when cells are exposed to oxidative stresses. This suggests that the enzyme plays a role in the cellular response to oxidative stresses, including reperfusion injury and aging (Kim et al., 2004).	Cytoplasm
**Glutamate receptors**			
18	GRIA1	GRIA1 is a subunit of the ionotropic glutamate receptor, AMPA, which acts as an excitatory glutamate receptor that modulates neuronal excitability in the CNS and mediates excitotoxic neuronal death (Shen and Limon, 2021).	Cell membrane, Endoplasmic reticulum membrane, Postsynaptic density membrane, Dendritic spine
19	GRM2	*Grm2* encodes for metabotropic glutamate receptor 2 (mGluR2), which has a more modulatory role in fine-tuning synaptic efficacy (Kew and Kemp, 2005).	cell membranes, Dendrite
20	CNIH2	In the brain, AMPA receptors assemble with several auxiliary subunits; CNIH2 is one of them; it binds to AMPA receptors and is important for AMPA receptor forward trafficking to synapses. CNIH2 modulates AMPA receptor biophysical properties by slowing receptor deactivation and desensitization kinetics (Gu et al., 2016).	Endoplasmic reticulum membrane, Postsynaptic density membrane, and Dendritic spine
21	SHISA6	SHISA6 is a single transmembrane protein that acts as a stable and directly interacting AMPA receptor auxiliary subunit. SHISA6 keeps AMPA receptors activated in the presence of glutamate, preventing full desensitization and synaptic depression (Klaassen et al., 2016).	Postsynaptic density membrane, dendritic spine
γ**-aminobutyric acid (GABA) receptors**			
22	GABRG2	GABRG2 encoding the γ-aminobutyric acid (GABA) A receptor gamma 2 (GABA_A_γ2) subunit is associated with genetic epilepsy. GABA_A_γ2 is decreased in the striatum and spinal cord of the MCAO group compared with the normal group. After acupuncture, the expression of GABA_A_γ2 is increased (Xu et al., 2015).	Postsynaptic density membrane
23	GABRB1	GABRB1 encoding GABA A receptor subunit beta 1 (GABA_A_β1) is a subunit involved in inhibitory effect on neurotransmission. Its disruption has been implicated in autism, and GABRB1 protein levels increased in the folic acid (FA)-treated cells in a concentration-dependent manner (Vasquez et al., 2013).	Postsynaptic density membrane
**Actin-binding proteins**			
24	EPB41L3	EPB41L3 is an important membrane cytoskeletal protein that confers stability and flexibility to erythrocytes through interactions with the cytoskeletal proteins spectrin and F-actin (Walensky et al., 1998).	cytoskeleton
25	TAGLN	TAGLN has been shown to have anti-inflammatory function in vascular smooth muscle cells through deactivation of ROS-mediated NF-κB pathways (Prasad et al., 2012).	Cytoplasm
26	MYL9	MYL9 is a regulatory subunit of the force-producing ATPase non-myosin II (NMII) and may regulate muscle contraction by regulating ATPase activity in the myosin head. It binds to actin filaments to control cytoskeletal dynamics and is subsequently involved in cell shape establishment, migration, polarity, adhesion, and signal-mechanical transduction (Lv et al., 2022).	Cytoplasm
**Energy metabolism**			
27	MT-CO2	MT-CO2 is a component of cytochrome c oxidase, which is the terminal enzyme complex of the respiratory chain, catalyzing the reduction of oxygen to water. Lack of MT-CO2 precludes assembly of cytochrome c oxidase beyond the first intermediate stage and leads to the degradation of unassembled subunits by the mitochondrial proteolytic system (Rossmanith et al., 2008).	mitochondrion
**Acute-phase proteins**			
28	ORM1	ORM1 is an acute-phase protein and plays an important role in inflammation and ischemic stroke. ORM1 performs various activities, acting as an acute-phase reactant and disease marker, regulating immunity, maintaining the capillary barrier function, regulating sphingomyelin metabolism, and scavenging ROS (Cheng et al., 2020).	Secreted in the extracellular space
29	SESN3	SESN3 is a stress-inducible protein. It is a strong genetic risk factor that regulates proconvulsive cytokines and genes and plays a key role in metabolic homeostasis. It has been reported that SESN3 regulates human hippocampal epilepsy (Shi et al., 2020).	Cytoplasm
**Cell signaling**			
30	PDE1B/1C	Phosphodiesterase enzyme (PDE) is a calcium- and calmodulin-dependent phosphodiesterase and limits the intracellular levels of cyclic nucleotides by catalyzing the hydrolysis of cAMP and cGMP. PDE1B and PDE1C are isoforms in PDE enzyme superfamily. PDE1B inhibition significantly enhances exosome release from microglia and protects neuronal cells against ischemic damage (Zang et al., 2020).	Cytoplasm
31	PDE10A	PDE10A is highly expressed in the basal ganglia or striatum. PDE10A degrades the intracellular second messengers cyclic adenosine monophosphate (cAMP) and cyclic guanosine monophosphate (cGMP) and terminates intracellular signaling that leads to the activation of transcription factor cAMP-responsive element-binding protein (CREB). Inhibition of PDE10A improved recovery of function after striatal stroke (Birjandi et al., 2021).	Cytoplasm
32	PRKCG	PRKCG is a member of the conventional protein kinase C (PKC) subfamily, requires Ca^2+^ for its activation, and is expressed only in the central nervous system. PRKCG functions initially in a deleterious fashion in response to ischemia, and later as a protective factor in the period of postischemic reperfusion (Chou and Messing, 2005).	Cytoplasm, Cell membrane, Dendrite
33	PRKAG2	PRKAG2 is the AMP-activated protein kinase (AMPK) non-catalytic subunit gamma 2. AMPK is an important energy-sensing enzyme that plays a key role in regulating cellular energy metabolism and functions by inactivating key enzymes involved in regulating *de novo* biosynthesis of fatty acid and cholesterol (Mo et al., 2019).	Nucleus, Cytoplasm
34	DGKG	DGKG is a member of the diacylglycerol kinase family and converts diacylglycerol (DAG) into phosphatidic acid (PA) and regulates the respective levels of these two bioactive lipids. PA is a pleiotropic lipid and plays various roles as a second messenger, such as cell proliferation, vesicle membrane trafficking, and cytoskeletal organization. DGKG is dominantly expressed in somatostatin (SST)-expressing GABAergic interneurons to regulate neurite outgrowth (Fukumoto et al., 2018).	Cell membrane, Cytoplasm, Cytoskeleton
35	ADCY5	Adenylate cyclase 5 (ADCY5) belongs to the adenylate cyclase family and can convert adenosine triphosphate (ATP) into the second messenger cAMP (Defer et al., 2000).	cell membrane
36	GNAL	GNAL is the stimulatory α subunit of the heterotrimeric G protein Golf that activates adenylate cyclase, thereby serving as a crucial mediator of intracellular signaling involved in olfaction and basal ganglia function (Yano et al., 2017).	

**Figure 4:**
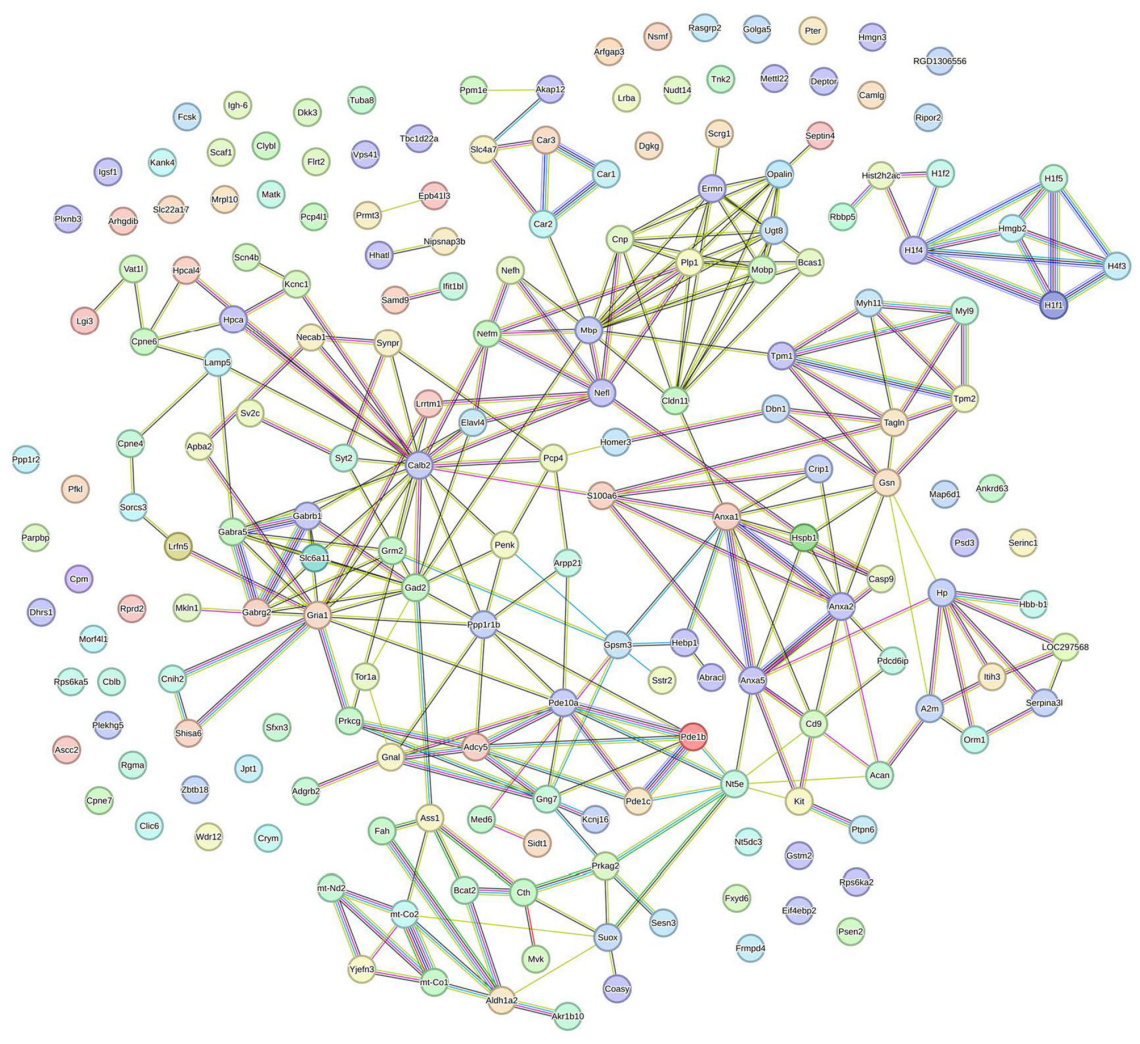
The interaction network of the 191 DEPs between the model and DZXI 7-day treatment groups (acquired with STRING). Colored nodes represent query proteins, edges represent protein-protein associations, and different color lines indicate the different interactions between proteins.

**Figure 5:**
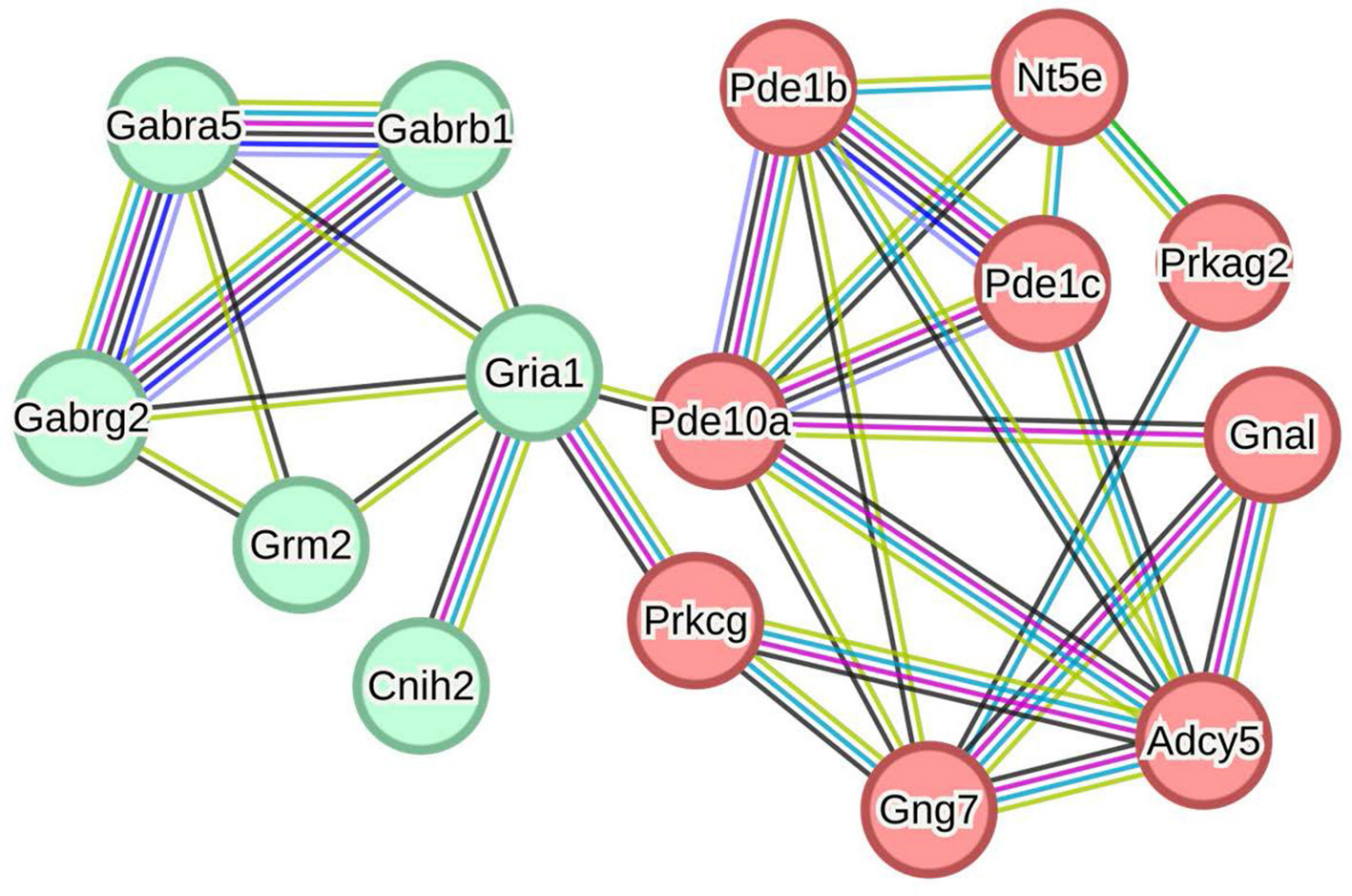
The interaction subnetwork of cellular signaling and chemical synaptic transmission associated proteins (acquired with STRING). Nodes in red represent proteins associated with cell signaling, and green represents chemical synaptic transmission. Pde1b, Calcium/calmodulin-dependent3′5′-cyclic nucleotide phosphodiesterase 1B; Pde1c, Calcium/calmodulin-dependent3′5′-cyclic nucleotide phosphodiesterase 1C; Pde10a, cAMP and cAMP-inhibited cGMP3′5′-cyclic phosphodiesterase 10A; Adcy5, Adenylate cyclase 5; Gnal, Guanine nucleotide-binding protein G(olf) subunit alpha; Gng7, Guanine nucleotide-binding protein subunit gamma; Nt5e, 5′-nucleotidase; Prkcg, Protein kinase C gamma type; Prkag2, Protein kinase AMP-activated non-catalytic subunit gamma 2; Gria1, Glutamate receptor 1; Grm2, Metabotropic glutamate receptor 2; Cnih2, Protein cornichon homolog 2; Gabra5, Gamma-aminobutyric acid receptor subunit alpha-5; Gabrg2, Gamma-aminobutyric acid receptor subunit gamma-2; Gabrb1, Gamma-aminobutyric acid receptor subunit beta-1.

STRING analysis was performed to construct a protein-protein interaction (PPI) network. One hundred eighty-eight DEPs between the MCAO model and DZXI groups were effectively recognized for PPI network analysis based on the STRING database to build a high-quality PPI network. The comprehensive PPI regulation network is shown in Figure 4. This network contains 188 proteins and 276 connected edges, with an average node degree of 2.94, and the average local clustering coefficient is 0.388, indicating that the relationship between nodes in this network is relatively close. We further extracted the specified function cluster network focused on cell signaling and chemical synaptic transmission (Figure 5). The functional sub-network is generated from the 15 DEPs, and nine of them are involved in cell signaling, while six of them are relevant to chemical synaptic transmission. This sub-network comprises 15 nodes and 31 edges, its average node degree is 4.13, and the average local clustering coefficient is 0.642, representing the verified complicated and tight interactions between these proteins. The network analysis proves that DZXI may regulate cell signaling and synaptic transmission during IS pathological processes.

### Molecular docking simulation validated the interaction between DZXI compounds and their targets *in silico*

**Figure 6:**
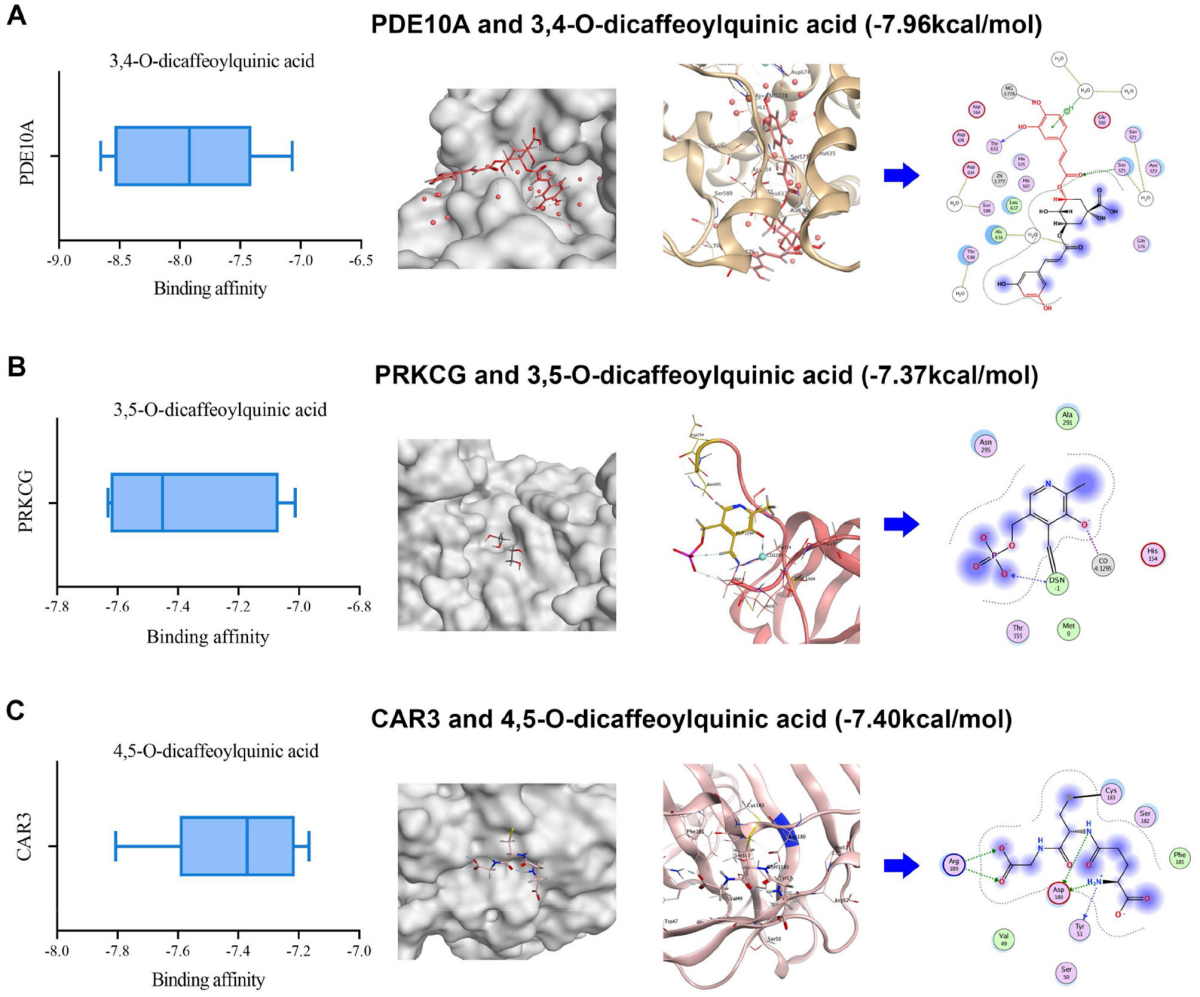
3D structural visualization of molecular docking simulation between DZXI compounds and their potentially targeted proteins. The binding affinity, binding sites (local and enlarged in 3D), and interacting residues (in 2D) of three target-compound pairs are exhibited. **(A)** PDE10A and 3,4-O-dicaffeoylquinic acid; **(B)** PRKCG and 3,5-O-dicaffeoylquinic acid; **(C)** CAR3 and 4,5-O-dicaffeoylquinic acid.

To calculate potential binding capacity between five DZXI active compounds [including scutellarin, 3,4-O-dicaffeoylquinic acid, 3,5-O-dicaffeoylquinic acid, 4,5-Odicaffeoylquinic acid, erigoster B which are highly exposed systematically *in vivo* (Guo et al., 2018; Huang et al., 2019)] and key target proteins, we performed computational simulations for each combination, respectively. The structures for each compound and protein were collected from the corresponding database. AutoDock was applied to conduct molecular virtual docking and calculate binding free energy. The binding energy was used as the reference to predict drug-target binding affinity. It is generally believed that a binding energy <0 indicates that tested molecules prefer to form a stable complex from spontaneous binding. Moreover, the lower binding energy represents a more stable complex formed and a greater possibility of combination occurring. Generally, the binding energy below −5 kcal/mol is considered a moderately tight docking affinity (Galma et al., 2021). Furthermore, a prior work using virtual screening to find a natural inhibitor for cAMP and cAMP-inhibited cGMP3′5′-cyclic phosphodiesterase 10A (PDE10A) accepted binding energy lower than−6 kcal/mol as sufficient affinity for the target (Al-Nema et al., 2018). Therefore, the present simulation indicated that each key target protein and DZXI active compound can form a relatively stable complex, because all their binding energy are negative and < -5 kcal/mol (Supplementary Table S3). Moreover, the proteins with the most compact binding affinity for scutellarin, 3,4-O-dicaffeoylquinic acid, 3,5-O-dicaffeoylquinic acid, 4,5-O-dicaffeoylquinic acid, and erigoster B are glutamate receptor 1 (GRIA1), PDE10A, protein kinase C gamma type (PRKCG), carbonic anhydrase 3 (CAR3), and GRIA1, respectively (Supplementary Table S3). It suggested the potential for high interaction of cell signaling-related proteins PDE10A and PRKCG, along with the chemical synaptic transmission-related protein GRIA1, with DZXI compounds. The visualization of their possible binding sites is shown in Figure 6.

## Discussion

Our study demonstrated that DZXI could alleviate neurological impairment, reduce ischemic infarction, and increase the survival of neural cells around the ischemic core in MCAO rats. Based on verifying the therapeutic effect of DZXI on focal cerebral ischemia, proteomic measurements were conducted on normal or ischemic cerebral hemispheres of rats in each group, followed by bioinformatics and molecular docking analysis. One hundred forty-five DEPs and 191 DEPs were involved in pathological progression and the DZXI therapeutic process. Pathological DEPs are enriched in oxidative stress response and response to wounding, reflecting physiological changes induced by ischemic injury, whereas DZXI therapeutic DEPs are enriched in modulation of trans-synaptic signaling and chemical synaptic transmission, which may indicate biological progress influenced by DZXI. Thirty-nine principal DEPs enrolled in concrete analysis were divided into 13 categories according to their specific functions. Moreover, cell signaling and chemical synaptic transmission, containing the most DEPs, were the most affected physiological processes under DZXI treatment. Molecular docking results suggested that a strong potential binding existed between cell signaling (PDE10A and PRKCG) or chemical synaptic transmission (GRIA1) related proteins and active compounds of DZXI. These results indicate that the multi-component characteristic of DZXI endows it with the ability to display therapeutic effects through multiple target proteins and pathways.

Previous pharmacological experiments indicated that DZXI and its main components, including caffeic acid ester fraction and scutellarin (Tu et al., 2021) had multiple beneficial effects (Wang J. et al., 2017; Li et al., 2017; Wang and Ma, 2018). Scutellarin has been proven to regulate nitric oxide (NO) production (Liu et al., 2005) and inhibiting cytotoxicity (Hong and Liu, 2004; Hu et al., 2005) induced by hypoxia to rescue neuronal damage. Moreover, caffeic acid ester fraction could inhibit microglial activation to provide neuroprotection against ischemic brain damage (Wang et al., 2012). Particularly, 4,5-O-dicaffeoylquinic acid and scutellarin have been reported to simultaneously regulate inhibitory and excitatory neurotransmitters as well as their receptors, including glutamatergic and GABAergic neuron synapses (Sheng et al., 2020). These findings suggest that caffeic acid ester and scutellarin may build the material foundation for DZXI therapeutic protection on cerebral ischemia.

In this study, the results integrated from TMT-based proteomic and molecular docking analysis potentially offered some novel potential targets for DZXI. Among these DEPs, several cell signaling and chemical synaptic transmission-related proteins were emphasized. PDE1B, PDE1C, and PDE10A, as cell signaling members which belong to the phosphodiesterases (PDEs) family, were detected in proteomic analysis, and their expression levels were significantly reduced after DZXI treatment. Further, the combined high binding affinity between PDE10A and 3,4-O-dicaffeoylquinic acid suggested a potential role of PDEs in DZXI treatment. PDEs degrade intracellular second messenger adenosine 3′,5′-cyclic monophosphate (cAMP) and/or guanosine 3′,5′-cyclic monophosphate (cGMP) to terminate intracellular signaling transmission. Prior studies have shown that cAMP/cGMP-mediated signals can activate downstream transcription factor cAMP response element-binding protein (CREB; Lee, 2015) and further induce CREB-regulated gene expression of BDNF to promote recovery after stroke (Caracciolo et al., 2018). Inhibition of striatal-specific PDE10A (Fujishige et al., 1999) could enhance BDNF expression by elevating cAMP and/or cGMP levels in the striatum (Giampà et al., 2010). Furthermore, cAMP-activated protein kinase (PKA), which is activated by cAMP and phosphorylates downstream CREB, might also mediate in this pathway (Carlezon et al., 2005). Another anti-IS drug, β-Caryophyllene, ameliorates cognitive impairment after IS through the cAMP/PKA/CREB/BDNF pathway in MCAO mice (Chen et al., 2020). Previous evidence collectively implies that the PDE/cAMP/PKA/CREB/BDNF pathway could be intimately involved in resisting ischemic injury. Moreover, recovery enhancement after striatal stroke induced by PDE10A inhibitor has already been confirmed in existing research (Birjandi et al., 2021; Beker et al., 2022a,b). The present study also identified PDEs reduction in broad proteomic profiling and predicted their binding affinities with DZXI compounds. Here, we suggest that DZXI promotes ischemic recovery by inhibiting PDE and activating related pathways like other PDE inhibitors; however, it should be further validated at the biochemical level in future research.

Another protein involved in cell signaling, protein kinase C gamma type (PRKCG), is a calcium-dependent enzyme of the PKC subfamily expressed only in the central nervous system (CNS; Tanaka and Nishizuka, 1994). PKC activation is implicated in the control of many vital brain functions, including synaptic plasticity, excitability, growth, proliferation, and apoptosis (Battaini, 2001). PRKCG null mice develop smaller infarcts than wild-type mice after permanent MCAO surgery and larger infarcts after transient MCAO (Aronowski et al., 2000; Aronowski and Labiche, 2003). These prior works suggest that PRKCG functions deleteriously in response to ischemia initially, and later as a protective factor in the period of postischemic reperfusion. Our proteomic detection also found the expression change triggered by DZXI of PRKCG. The strong binding affinity predicted the interaction between PRKCG and 3, 5-O-dicaffeoylquinic acid, suggesting PRKCG as a potential therapeutic target for DZXI at the post-ischemic later stage. However, the mechanism under PRKCG that acts as the protective factor against reperfusion injury needs more exploration.

Furthermore, elevated level of some chemical synaptic transmission-related proteins was also detected in our proteomic profiling, such as GRIA1 and CNIH2, SHISA6, which are the constituent and auxiliary subunits of Alpha-amino-3-hydroxy-5-methyl-4-isoxazole propionic acid receptors (AMPARs). AMPARs are principal postsynaptic ionotropic glutamate receptors that mediate excitatory synaptic transmission in the CNS. Whereas, a previous study has shown that low mRNA level of GRIA1 is associated with atherosclerosis in vascular smooth muscle cells (Gallina et al., 2021). Furthermore, the PKA phosphorylation of GRIA1 residue is considered a necessary pre-requisite step in synaptic trafficking of GluA1-containing AMPARs during long-term potentiation (LTP; Esteban et al., 2003). Indeed, the decrease in hippocampal LTP intensity in mice experiencing ischemic injury and spatial impairment was observed in prior research (Li et al., 2013). Therefore, combining predicted interaction between GRIA1 and DZXI active compounds, we inferred that DZXI might modulate GRIA1 expression level or even phosphorylation state in LTP maintenance to prevent ischemic damage, though further direct experimental evidence is required in the future.

**Figure 7:**
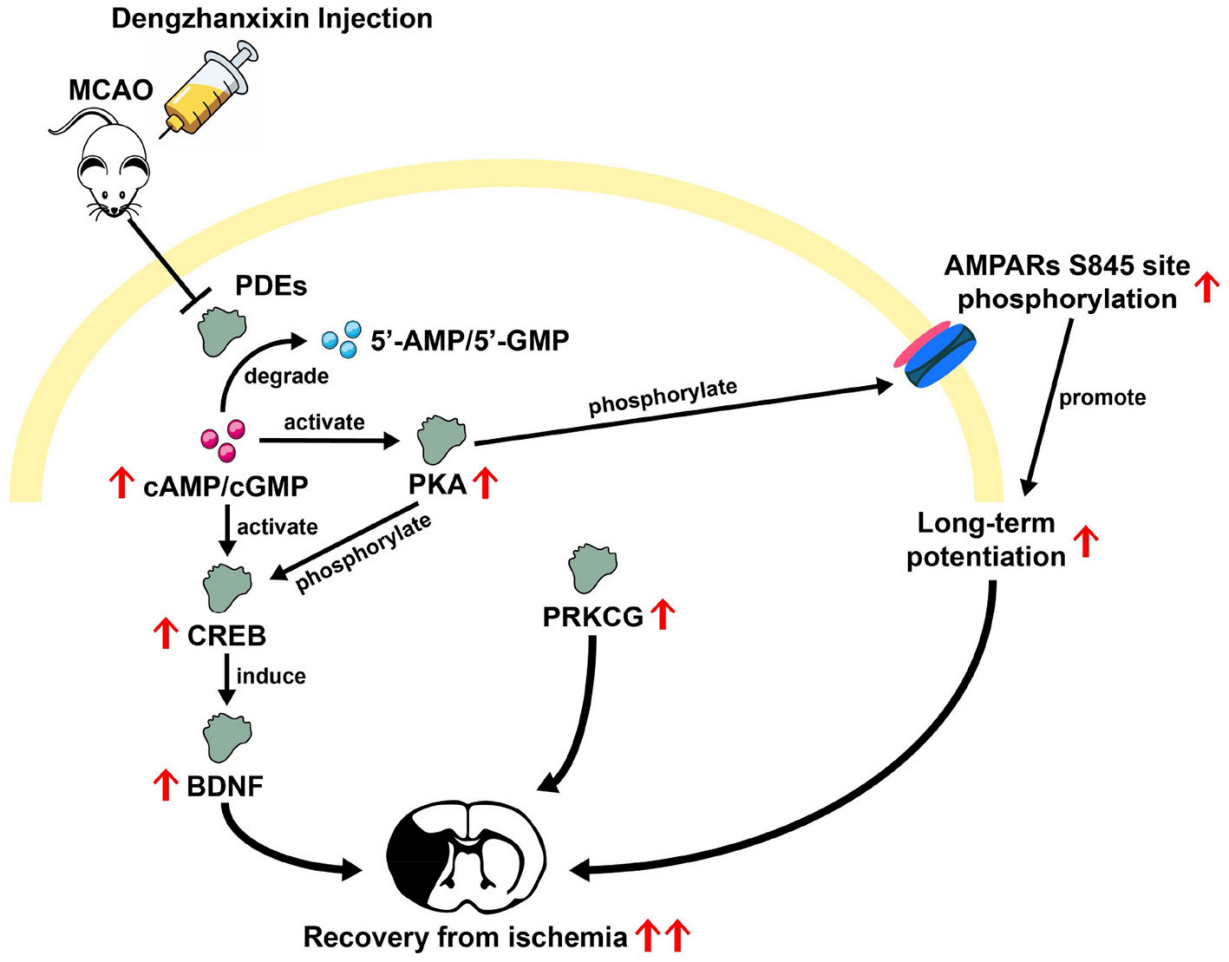
Overview of possible mechanisms inferred from prior studies underlying the DZXI therapeutic effect on ischemic stroke.

Neuroprotective agents represent a promising adjunctive strategy to complement vascular recanalization treatment. This therapeutic paradigm necessitates the development of novel neuroprotective targets with enhanced efficacy and safety for IS. Based on broad proteomic exploration and molecular docking prediction, our findings suggested that DZXI intervention improves functional recovery and confers neuroprotective effects by modulating multiple previously unidentified potential targets, such as PDE10A, PRKCG, and GRIA1. The cumulative evidence from prior studies provides a mechanistic rationale for these newly identified potential targets of DZXI in promoting restoration from ischemia. The overview of the proposed multi-target mechanism under DZXI therapy is shown in Figure 7. However, the present investigation offers multi-dimensional evidence to substantially expand known therapeutic targets of DZXI and suggests their potential mechanisms; further experimental validation is needed. Therefore, there are several limitations in this study that should be addressed in the future. First, quantitative experiments on specific proteins or biochemical molecules are required to directly verify a plausible pathway regulated by DZXI. Second, the multi-component characteristic of DZXI confers a multi-target and multi-pathway synergy effect. Besides the multi-functionality of DZXI, it also brings more difficulties in untangling its potential mechanistic pathway clearly and thoroughly. Consequently, advanced investigations utilizing animal models are warranted to illustrate the explicit effect and precise underlying mechanism for each main compound in DZXI. Finally, the preclinical optimization studies systematically investigating the therapeutic efficacy of individual compounds in DZXI or their different combinations will facilitate finding a suitable intervention for IS patients.

## Conclusion

In summary, our results demonstrated that DZXI intervention ameliorated neurological deficits and offered neuroprotection to neural cells in the ischemic penumbra. Multiple DEPs identified through broad proteomic profiling combined with bioinformatics analysis suggested that modulation of trans-synaptic signaling transmission was partly influenced by DZXI. Moreover, several potential target proteins of DZXI related to cell signaling and synaptic chemical transmission, including PDE10A, PRKCG, and GRIA1, were predicted to have a high binding affinity to DZXI main compounds. Multi-component in DZXI implied the complexity of the target protein composition, ensuring its therapeutic outcomes. Although the precise pathway underlying DZXI's therapeutic effect needs more exploration, the evidence in our study is sufficient to support that DZXI treatment has the potential to become a valuable adjunctive therapy for IS.

## Data Availability

The original data presented in this study is available in a public repository and it can be found through http://proteomecentral.proteomexchange.org/cgi/GetDataset?ID=PXD026918.

## References

[B1] Al-Nema M. Gaurav A. Akowuah G. (2018). Discovery of natural product inhibitors of phosphodiesterase 10A as novel therapeutic drug for schizophrenia using a multistep virtual screening. Comput. Biol. Chem. 77, 52–63. 10.1016/j.compbiolchem.2018.09.001 30240986

[B2] An H. Tao W. Liang Y. Li P. Li M. Zhang X.. (2021). Dengzhanxixin injection ameliorates cognitive impairment through a neuroprotective mechanism based on mitochondrial preservation in patients with acute ischemic stroke. Front. Pharmacol. 12:712436. 10.3389/fphar.2021.712436 34526899 PMC8435665

[B3] Aronowski J. Grotta J. C. Strong R. Waxham M. N. (2000). Interplay between the gamma isoform of PKC and calcineurin in regulation of vulnerability to focal cerebral ischemia. J. Cereb. Blood Flow Metab. 20, 343–349. 10.1097/00004647-200002000-00016 10698072

[B4] Aronowski J. Labiche L. A. (2003). Perspectives on reperfusion-induced damage in rodent models of experimental focal ischemia and role of gamma-protein kinase C. ILAR J. 44, 105–109. 10.1093/ilar.44.2.105 12652005

[B5] Battaini F. (2001). Protein kinase C isoforms as therapeutic targets in nervous system disease states. Pharmacol. Res. 44, 353–361. 10.1006/phrs.2001.0893 11712865

[B6] Bederson J. B. Pitts L. H. Germano S. M. Nishimura M. C. Davis R. L. Bartkowski H. M. (1986). Evaluation of 2,3,5-triphenyltetrazolium chloride as a stain for detection and quantification of experimental cerebral infarction in rats. Stroke 17, 1304–1308. 10.1161/01.STR.17.6.1304 2433817

[B7] Beker M. C. Caglayan A. B. Altunay S. Ozbay E. Ates N. Kelestemur T.. (2022a). Phosphodiesterase 10A is a critical target for neuroprotection in a mouse model of ischemic stroke. Mol. Neurobiol. 59, 574–589. 10.1007/s12035-021-02621-5 34735672

[B8] Beker M. C. Pence M. E. Yagmur S. Caglayan B. Caglayan A. Kilic U.. (2022b). Phosphodiesterase 10A deactivation induces long-term neurological recovery. Peri-infarct remodeling and pyramidal tract plasticity after transient focal cerebral ischemia in mice. Exp. Neurol. 358:114221. 10.1016/j.expneurol.2022.114221 36075453

[B9] Birjandi S. Z. Abduljawad N. Nair S. Dehghani M. Suzuki K. Kimura H.. (2021). Phosphodiesterase 10A inhibition leads to brain region-specific recovery based on stroke type. Transl. Stroke Res. 12, 303–315. 10.1007/s12975-020-00819-8 32378029 PMC7644574

[B10] Boersma H. H. Kietselaer B. L. Stolk L. M. Bennaghmouch A. Hofstra L. Narula J.. (2005). Past, present, and future of annexin A5: from protein discovery to clinical applications. J. Nucl. Med. 46, 2035–2050. 16330568

[B11] Boggs J. M. (2006). Myelin basic protein: a multifunctional protein. Cell. Mol. Life Sci. 63, 1945–1961. 10.1007/s00018-006-6094-7 16794783 PMC11136439

[B12] Bosio A. Binczek E. Le Beau M. M. Fernald A. A. Stoffel W. (1996). The human gene CGT encoding the UDP-galactose ceramide galactosyl transferase (cerebroside synthase): cloning, characterization, and assignment to human chromosome 4, band q26. Genomics 34, 69–75. 10.1006/geno.1996.0242 8661025

[B13] Burley S. K. Berman H. M. Kleywegt G. J. Markley J. L. Nakamura H. Velankar S. (2017). Protein data bank (PDB): the single global macromolecular structure archive. Methods Mol. Biol. 1607, 627–641. 10.1007/978-1-4939-7000-1_26 28573592 PMC5823500

[B14] Campbell B. C. V. De Silva D. A. Macleod M. R. Coutts S. B. Schwamm L. H. Davis S. M.. (2019). Ischaemic stroke. Nat Rev Dis Primers 5:70. 10.1038/s41572-019-0118-8 31601801

[B15] Campbell B. C. V. Khatri P. (2020). Stroke. Lancet 396, 129–142. 10.1016/S0140-6736(20)31179-X 32653056

[B16] Caracciolo L. Marosi M. Mazzitelli J. Latifi S. Sano Y. Galvan L.. (2018). CREB controls cortical circuit plasticity and functional recovery after stroke. Nat. Commun. 9:2250. 10.1038/s41467-018-04445-9 29884780 PMC5993731

[B17] Carlezon W. A. Jr. Duman R. S. Nestler E. J. (2005). The many faces of CREB. Trends Neurosci. 28, 436–445. 10.1016/j.tins.2005.06.005 15982754

[B18] Chai L. Guo H. Li H. Wang S. Wang Y. L. Shi F.. (2013). Scutellarin and caffeic acid ester fraction, active components of dengzhanxixin injection, upregulate neurotrophins synthesis and release in hypoxia/reoxygenation rat astrocytes. J. Ethnopharmacol. 150, 100–107. 10.1016/j.jep.2013.08.011 24012966

[B19] Chen J. Li Y. Wang L. Zhang Z. Lu D. Lu M.. (2001). Therapeutic benefit of intravenous administration of bone marrow stromal cells after cerebral ischemia in rats. Stroke 32, 1005–1011. 10.1161/01.STR.32.4.1005 11283404

[B20] Chen S. Wang Y. Wang X. He M. Zhang L. Dong Z. (2020). PKA-dependent membrane surface recruitment of CI-AMPARs is crucial for BCP-mediated protection against post-acute ischemic stroke cognitive impairment. Front. Neurol. 11:566067. 10.3389/fneur.2020.566067 33391143 PMC7772322

[B21] Cheng X. Liu D. Xing R. Song H. Tian X. Yan C.. (2020). Orosomucoid 1 attenuates doxorubicin-induced oxidative stress and apoptosis in cardiomyocytes via Nrf2 signaling. Biomed Res. Int. 2020:5923572. 10.1155/2020/5923572 33134382 PMC7591952

[B22] Chou W. H. Messing R. O. (2005). Protein kinase C isozymes in stroke. Trends Cardiovasc. Med. 15, 47–51. 10.1016/j.tcm.2005.01.003 15885569

[B23] Commission N. P. (2005). Pharmacopoeia of the People's Republic of China. Beijing: Chemical Industry Press.

[B24] Dallacasagrande V. Hajjar K. A. (2020). Annexin A2 in inflammation and host defense. Cells 9:1499. 10.3390/cells9061499 32575495 PMC7348701

[B25] Defer N. Best-Belpomme M. Hanoune J. (2000). Tissue specificity and physiological relevance of various isoforms of adenylyl cyclase. Am. J. Physiol. Renal Physiol. 279, F400–F416. 10.1152/ajprenal.2000.279.3.F400 10966920

[B26] Ding R. F. Li Z. X. (2009). The clinical application of breviscapine preparations. Tianjin Pharmacy 21, 60–63. 10.3969/j.issn.1006-5687.2009.02.031

[B27] Donkervoort S. Mohassel P. Laugwitz L. Zaki M. S. Kamsteeg E. J. Maroofian R.. (2020). Biallelic loss of function variants in SYT2 cause a treatable congenital onset presynaptic myasthenic syndrome. Am. J. Med. Genet. A 182, 2272–2283. 10.1002/ajmg.a.61765 32776697 PMC7959540

[B28] Elitt M. S. Barbar L. Shick H. E. Powers B. E. Maeno-Hikichi Y. Madhavan M.. (2020). Suppression of proteolipid protein rescues pelizaeus-merzbacher disease. Nature 585, 397–403. 10.1038/s41586-020-2494-3 32610343 PMC7810164

[B29] Esteban J. A. Shi S. H. Wilson C. Nuriya M. Huganir R. L. Malinow R. (2003). PKA phosphorylation of AMPA receptor subunits controls synaptic trafficking underlying plasticity. Nat. Neurosci. 6, 136–143. 10.1038/nn997 12536214

[B30] Fluri F. Schuhmann M. K. Kleinschnitz C. (2015). Animal models of ischemic stroke and their application in clinical research. Drug Des. Devel. Ther. 9, 3445–3454. 10.2147/DDDT.S56071 26170628 PMC4494187

[B31] Fujishige K. Kotera J. Omori K. (1999). Striatum- and testis-specific phosphodiesterase PDE10A isolation and characterization of a rat PDE10A. Eur. J. Biochem. 266, 1118–1127. 10.1046/j.1432-1327.1999.00963.x 10583409

[B32] Fukumoto K. Tamada K. Toya T. Nishino T. Yanagawa Y. Takumi T. (2018). Identification of genes regulating GABAergic interneuron maturation. Neurosci. Res. 134, 18–29. 10.1016/j.neures.2017.11.010 29203264

[B33] Gallina A. L. Rykaczewska U. Wirka R. C. Caravaca A. S. Shavva V. S. Youness M.. (2021). AMPA-type glutamate receptors associated with vascular smooth muscle cell subpopulations in atherosclerosis and vascular injury. Front Cardiovasc Med 8:655869. 10.3389/fcvm.2021.655869 33959644 PMC8093397

[B34] Galma W. Endale M. Getaneh E. Eswaramoorthy R. Assefa T. Melaku Y. (2021). Antibacterial and antioxidant activities of extracts and isolated compounds from the roots extract of Cucumis prophetarum and *in silico* study on DNA gyrase and human peroxiredoxin 5. BMC Chem. 15:32. 10.1186/s13065-021-00758-x 33957962 PMC8103605

[B35] GBDS Collaborators. (2021). Global, regional, and national burden of stroke and its risk factors, 1990–2019: a systematic analysis for the Global Burden of Disease Study 2019. Lancet Neurol. 20, 795–820. 10.1016/S1474-4422(21)00252-0 34487721 PMC8443449

[B36] George P. M. Steinberg G. K. (2015). Novel stroke therapeutics: unraveling stroke pathophysiology and its impact on clinical treatments. Neuron 87, 297–309. 10.1016/j.neuron.2015.05.041 26182415 PMC4911814

[B37] Giampà C. Laurenti D. Anzilotti S. Bernardi G. Menniti F. S. Fusco F. R. (2010). Inhibition of the striatal specific phosphodiesterase PDE10A ameliorates striatal and cortical pathology in R6/2 mouse model of Huntington's disease. PLoS ONE 5:e13417. 10.1371/journal.pone.0013417 20976216 PMC2955524

[B38] Gu X. Mao X. Lussier M. P. Hutchison M. A. Zhou L. Hamra F. K.. (2016). GSG1L suppresses AMPA receptor-mediated synaptic transmission and uniquely modulates AMPA receptor kinetics in hippocampal neurons. Nat. Commun. 7:10873. 10.1038/ncomms10873 26932439 PMC4778064

[B39] Guo X. Lin S. Yang P. Ye J. Du J. Mu X.. (2018). Rapid characterization and identification of the chemical constituents and rat metabolites of deng-zhan-xi-xin injection using ultra high performance liquid chromatography coupled with quadrupole time-of-flight mass spectrometry. J. Sep. Sci. 41, 3569–3582. 10.1002/jssc.201800470 30062810

[B40] Hong H. Liu G. Q. (2004). Protection against hydrogen peroxide-induced cytotoxicity in PC12 cells by scutellarin. Life Sci. 74, 2959–2973. 10.1016/j.lfs.2003.09.074 15051420

[B41] Hu X. M. Zhou M. M. Hu X. M. Zeng F. D. (2005). Neuroprotective effects of scutellarin on rat neuronal damage induced by cerebral ischemia/reperfusion. Acta Pharmacol. Sin. 26, 1454–1459. 10.1111/j.1745-7254.2005.00239.x 16297343

[B42] Huang J. Su Y. Yang C. Li S. Wu Y. Chen B.. (2019). An integrated pharmacokinetic study of Dengzhanxixin injection in rats by combination of multicomponent pharmacokinetics and anti-myocardial ischemic assay. RSC Adv. 9, 25309–25317. 10.1039/C9RA03917A 35530075 PMC9070076

[B43] Huang J. Upadhyay U. M. Tamargo R. J. (2006). Inflammation in stroke and focal cerebral ischemia. Surg. Neurol. 66, 232–245. 10.1016/j.surneu.2005.12.028 16935624

[B44] Iida A. Saito S. Sekine A. Mishima C. Kitamura Y. Kondo K.. (2002). Catalog of 86 single-nucleotide polymorphisms (SNPs) in three uridine diphosphate glycosyltransferase genes: UGT2A1, UGT2B15, and UGT8. J. Hum. Genet. 47, 505–510. 10.1007/s100380200075 12376738

[B45] Iłżecki M. Iłżecka J. Przywara S. Terlecki P. Grabarska A. Stepulak A.. (2017). Effect of carotid endarterectomy on brain damage markers. Acta Neurol. Scand. 135, 352–359. 10.1111/ane.12607 27126899

[B46] Kelly K. K. MacPherson A. M. Grewal H. Strnad F. Jones J. W. Yu J.. (2016). Col1a1+ perivascular cells in the brain are a source of retinoic acid following stroke. BMC Neurosci. 17:49. 10.1186/s12868-016-0284-5 27422020 PMC4947279

[B47] Kew J. N. Kemp J. A. (2005). Ionotropic and metabotropic glutamate receptor structure and pharmacology. Psychopharmacology 179, 4–29. 10.1007/s00213-005-2200-z 15731895

[B48] Kida E. Palminiello S. Golabek A. A. Walus M. Wierzba-Bobrowicz T. Rabe A.. (2006). Carbonic anhydrase II in the developing and adult human brain. J. Neuropathol. Exp. Neurol. 65, 664–674. 10.1097/01.jnen.0000225905.52002.3e 16825953

[B49] Kim G. Lee T. H. Wetzel P. Geers C. Robinson M. A. Myers T. G.. (2004). Carbonic anhydrase III is not required in the mouse for normal growth, development, and life span. Mol. Cell. Biol. 24, 9942–9947. 10.1128/MCB.24.22.9942-9947.2004 15509796 PMC525481

[B50] Klaassen R. V. Stroeder J. Coussen F. Hafner A. S. Petersen J. D. Renancio C.. (2016). Shisa6 traps AMPA receptors at postsynaptic sites and prevents their desensitization during synaptic activity. Nat. Commun. 7:10682. 10.1038/ncomms10682 26931375 PMC4778035

[B51] Lalancette-Hébert M. Gowing G. Simard A. Weng Y. C. Kriz J. (2007). Selective ablation of proliferating microglial cells exacerbates ischemic injury in the brain. J. Neurosci. 27, 2596–2605. 10.1523/JNEUROSCI.5360-06.2007 17344397 PMC6672496

[B52] Lee D. (2015). Global and local missions of cAMP signaling in neural plasticity, learning, and memory. Front. Pharmacol. 6:161. 10.3389/fphar.2015.00161 26300775 PMC4523784

[B53] Lee J. Y. Kim G. Park S. Kang S. M. Jang Y. Lee S. H. (2015). Associations between genetic variants and angiographic characteristics in patients with coronary artery disease. J. Atheroscler. Thromb. 22, 363–371. 10.5551/jat.26047 25328121

[B54] Li J. G. Wang L. Q. Yang X. Y. Chen Z. Lai L. Y. W. Xu H.. (2017). Chinese herbal medicine dengzhan xixin injection for acute ischemic stroke: a systematic review and meta-analysis of randomised controlled trials. Complement. Ther. Med. 34, 74–85. 10.1016/j.ctim.2017.08.004 28917378

[B55] Li W. Huang R. Shetty R. A. Thangthaeng N. Liu R. Chen Z.. (2013). Transient focal cerebral ischemia induces long-term cognitive function deficit in an experimental ischemic stroke model. Neurobiol. Dis. 59, 18–25. 10.1016/j.nbd.2013.06.014 23845275 PMC3790570

[B56] Lin X. J. Wang M. P. Liu Q. H. (2003). Effects of dengzhan xixin injection on hemarheology of cerebral infarction patients. J. Clin. Neurol. 16, 375–375. 10.3969/j.issn.1004-1648.2003.06.025

[B57] Lin Y. H. (2020). A Quantitative Analysis Method of Multi-components With Single Marker for Dengzhanxixin Injection. Beijing: China National Intellectual Property Administration.

[B58] Lipton P. (1999). Ischemic cell death in brain neurons. Physiol. Rev. 79, 1431–1568. 10.1152/physrev.1999.79.4.1431 10508238

[B59] Liu H. Yang X. Tang R. Liu J. Xu H. (2005). Effect of scutellarin on nitric oxide production in early stages of neuron damage induced by hydrogen peroxide. Pharmacol. Res. 51, 205–210. 10.1016/j.phrs.2004.09.001 15661569

[B60] Lv M. Luo L. Chen X. (2022). The landscape of prognostic and immunological role of myosin light chain 9 (MYL9) in human tumors. Immun. Inflamm. Dis. 10, 241–254. 10.1002/iid3.557 34729929 PMC8767521

[B61] McBride M. W. Carr F. J. Graham D. Anderson N. H. Clark J. S. Lee W. K.. (2003). Microarray analysis of rat chromosome 2 congenic strains. Hypertension 41, 847–853. 10.1161/01.HYP.0000047103.07205.03 12624007

[B62] Mhairi Macrae I. (1992). New models of focal cerebral ischaemia. Br. J. Clin. Pharmacol. 34, 302–308. 10.1111/j.1365-2125.1992.tb05634.x 1457262 PMC1381409

[B63] Mimura J. Inose-Maruyama A. Taniuchi S. Kosaka K. Yoshida H. Yamazaki H.. (2019). Concomitant Nrf2- and ATF4-activation by carnosic acid cooperatively induces expression of cytoprotective genes. Int. J. Mol. Sci. 20:1706. 10.3390/ijms20071706 30959808 PMC6480217

[B64] Mo X. Zhang H. Zhou Z. Zhu Z. HuangFu X. Xu T.. (2019). SNPs rs10224002 in PRKAG2 may disturb gene expression and consequently affect hypertension. Mol. Biol. Rep. 46, 1617–1624. 10.1007/s11033-019-04610-3 30689184

[B65] Moskowitz M. A. Lo E. H. Iadecola C. (2010). The science of stroke: mechanisms in search of treatments. Neuron 67, 181–198. 10.1016/j.neuron.2010.07.002 20670828 PMC2957363

[B66] Namura S. Ooboshi H. Liu J. Yenari M. A. (2013). Neuroprotection after cerebral ischemia. Ann. N. Y. Acad. Sci. 1278, 25–32. 10.1111/nyas.12087 23488559 PMC3645884

[B67] Obrenovitch T. P. Urenjak J. Richards D. A. Ueda Y. Curzon G. Symon L. (1993). Extracellular neuroactive amino acids in the rat striatum during ischaemia: comparison between penumbral conditions and ischaemia with sustained anoxic depolarisation. J. Neurochem. 61, 178–186. 10.1111/j.1471-4159.1993.tb03553.x 8515264

[B68] Perestenko P. V. Pooler A. M. Noorbakhshnia M. Gray A. Bauccio C. Jeffrey McIlhinney R. A. (2010). Copines-1,−2,−3,−6 and−7 show different calcium-dependent intracellular membrane translocation and targeting. FEBS J. 277, 5174–5189. 10.1111/j.1742-4658.2010.07935.x 21087455

[B69] Powers W. J. Rabinstein A. A. Ackerson T. Adeoye O. M. Bambakidis N. C. Becker K.. (2019). Guidelines for the early management of patients with acute ischemic stroke: 2019 update to the 2018 guidelines for the early management of acute ischemic stroke: a guideline for healthcare professionals from the American Heart Association/American Stroke Association. Stroke 50, e344–e418. 10.1161/STR.0000000000000211 31662037

[B70] Prasad S. S. Russell M. Nowakowska M. Williams A. Yauk C. (2012). Gene expression analysis to identify molecular correlates of pre- and post-conditioning derived neuroprotection. J. Mol. Neurosci. 47, 322–339. 10.1007/s12031-012-9751-3 22467039

[B71] Purvis G. S. D. Solito E. Thiemermann C. (2019). Annexin-A1: therapeutic potential in microvascular disease. Front. Immunol. 10:938. 10.3389/fimmu.2019.00938 31114582 PMC6502989

[B72] Reinhard J. R. Kriz A. Galic M. Angliker N. Rajalu M. Vogt K. E.. (2016). The calcium sensor Copine-6 regulates spine structural plasticity and learning and memory. Nat. Commun. 7:11613. 10.1038/ncomms11613 27194588 PMC4874034

[B73] Rossmanith W. Freilinger M. Roka J. Raffelsberger T. Moser-Thier K. Prayer D.. (2008). Isolated cytochrome c oxidase deficiency as a cause of MELAS. J. Med. Genet. 45, 117–121. 10.1136/jmg.2007.052076 18245391

[B74] Sharma M. C. (2019). Annexin A2 (ANX A2): an emerging biomarker and potential therapeutic target for aggressive cancers. Int. J. Cancer 144, 2074–2081. 10.1002/ijc.31817 30125343

[B75] Shen K. Limon A. (2021). Transcriptomic expression of AMPA receptor subunits and their auxiliary proteins in the human brain. Neurosci. Lett. 755:135938. 10.1016/j.neulet.2021.135938 33915226 PMC8169612

[B76] Sheng N. Zheng H. Li M. Li M. Wang Z. Peng Y.. (2020). 4,5 caffeoylquinic acid and scutellarin, identified by integrated metabolomics and proteomics approach as the active ingredients of Dengzhan Shengmai, act against chronic cerebral hypoperfusion by regulating glutamatergic and GABAergic synapses. Pharmacol. Res. 152:104636. 10.1016/j.phrs.2020.104636 31926275

[B77] Shi Z. Lei Z. Wu F. Xia L. Ruan Y. Xu Z. C. (2020). Increased sestrin3 contributes to post-ischemic seizures in the diabetic condition. Front. Neurosci. 14:591207. 10.3389/fnins.2020.591207 33519354 PMC7843462

[B78] Sly W. S. Hu P. Y. (1995). Human carbonic anhydrases and carbonic anhydrase deficiencies. Annu. Rev. Biochem. 64, 375–401. 10.1146/annurev.bi.64.070195.002111 7574487

[B79] Stephan A. H. Barres B. A. Stevens B. (2012). The complement system: an unexpected role in synaptic pruning during development and disease. Annu. Rev. Neurosci. 35, 369–389. 10.1146/annurev-neuro-061010-113810 22715882

[B80] Szklarczyk D. Kirsch R. Koutrouli M. Nastou K. Mehryary F. Hachilif R.. (2023). The STRING database in 2023: protein-protein association networks and functional enrichment analyses for any sequenced genome of interest. Nucleic Acids Res. 51, D638–D646. 10.1093/nar/gkac1000 36370105 PMC9825434

[B81] Szydlowska K. Tymianski M. (2010). Calcium, ischemia and excitotoxicity. Cell Calcium 47, 122–129. 10.1016/j.ceca.2010.01.003 20167368

[B82] Tanaka C. Nishizuka Y. (1994). The protein kinase C family for neuronal signaling. Annu. Rev. Neurosci. 17, 551–567. 10.1146/annurev.ne.17.030194.003003 8210187

[B83] Trott O. Olson A. J. (2010). AutoDock Vina: improving the speed and accuracy of docking with a new scoring function, efficient optimization, and multithreading. J. Comput. Chem. 31, 455–461. 10.1002/jcc.21334 19499576 PMC3041641

[B84] Tu X. X. Tang Q. Zhao Y. J. Du J. Wu L. M. Dong Y. (2021). Simultaneous determination two types of substances in Dengzhan Xixin injection by quantitative analysis of multie-components with single marker and the external standard method. Chin. J. Pharm. Anal. 41, 798–808. 10.16155/j.0254-1793.2021.05.06

[B85] van Genderen H. O. Kenis H. Hofstra L. Narula J. Reutelingsperger C. P. (2008). Extracellular annexin A5: functions of phosphatidylserine-binding and two-dimensional crystallization. Biochim. Biophys. Acta 1783, 953–963. 10.1016/j.bbamcr.2008.01.030 18334229

[B86] Vasquez K. Kuizon S. Junaid M. Idrissi A. E. (2013). The effect of folic acid on GABA(A)-B 1 receptor subunit. Adv. Exp. Med. Biol. 775, 101–109. 10.1007/978-1-4614-6130-2_8 23392927

[B87] Walensky L. D. Shi Z. T. Blackshaw S. DeVries A. C. Demas G. E. Gascard P.. (1998). Neurobehavioral deficits in mice lacking the erythrocyte membrane cytoskeletal protein 4.1. Curr. Biol. 8, 1269–1272. 10.1016/S0960-9822(07)00536-2 9822582

[B88] Wallner B. P. Mattaliano R. J. Hession C. Cate R. L. Tizard R. Sinclair L. K.. (1986). Cloning and expression of human lipocortin, a phospholipase A2 inhibitor with potential anti-inflammatory activity. Nature 320, 77–81. 10.1038/320077a0 2936963

[B89] Wang F. J. Xie Y. M. Liao X. Jia M. (2015). Dengzhan xixin injection as an adjuvant treatment for angina pectoris: a systematic review and meta-analysis of randomized controlled trials. Zhongguo Zhong Yao Za Zhi 40, 3298–3307. 10.4268/cjcmm20151634 26790311

[B90] Wang J. Xie Y. Zhao S. Zhang J. Chai Y. Li Y.. (2017). Dengzhanxixin injection for cerebral infarction: a systematic review and meta-analysis of randomized controlled trials. Medicine 96:e7674. 10.1097/MD.0000000000007674 28796050 PMC5556216

[B91] Wang J. Yang Z. Liu C. Zhao Y. Chen Y. (2013). Activated microglia provide a neuroprotective role by balancing glial cell-line derived neurotrophic factor and tumor necrosis factor-alpha secretion after subacute cerebral ischemia. Int. J. Mol. Med. 31, 172–178. 10.3892/ijmm.2012.1179 23151666 PMC3573737

[B92] Wang L. Ma Q. (2018). Clinical benefits and pharmacology of scutellarin: a comprehensive review. Pharmacol. Ther. 190, 105–127. 10.1016/j.pharmthera.2018.05.006 29742480

[B93] Wang S. X. Guo H. Hu L. M. Liu Y. N. Wang Y. F. Kang L. Y.. (2012). Caffeic acid ester fraction from Erigeron breviscapus inhibits microglial activation and provides neuroprotection. Chin. J. Integr. Med. 18, 437–444. 10.1007/s11655-012-1114-y 22821656

[B94] Wang W. Jiang B. Sun H. Ru X. Sun D. Wang L.. (2017). Prevalence, incidence, and mortality of stroke in China: results from a nationwide population-based survey of 480 687 adults. Circulation 135, 759–771. 10.1161/CIRCULATIONAHA.116.025250 28052979

[B95] Wu S. Wu B. Liu M. Chen Z. Wang W. Anderson C. S.. (2019). Stroke in China: advances and challenges in epidemiology, prevention, and management. Lancet Neurol. 18, 394–405. 10.1016/S1474-4422(18)30500-3 30878104

[B96] Xu Q. Yang J. W. Cao Y. Zhang L. W. Zeng X. H. Li F.. (2015). Acupuncture improves locomotor function by enhancing GABA receptor expression in transient focal cerebral ischemia rats. Neurosci. Lett. 588, 88–94. 10.1016/j.neulet.2014.12.057 25556683

[B97] Yamatani H. Kawasaki T. Mita S. Inagaki N. Hirata T. (2010). Proteomics analysis of the temporal changes in axonal proteins during maturation. Dev. Neurobiol. 70, 523–537. 10.1002/dneu.20794 20225247

[B98] Yano H. Provasi D. Cai N. S. Filizola M. Ferré S. Javitch J. A. (2017). Development of novel biosensors to study receptor-mediated activation of the G-protein alpha subunits G(s) and G(olf). J. Biol. Chem. 292, 19989–19998. 10.1074/jbc.M117.800698 29042444 PMC5723988

[B99] Yenari M. A. Minami M. Sun G. H. Meier T. J. Kunis D. M. McLaughlin J. R.. (2001). Calbindin d28k overexpression protects striatal neurons from transient focal cerebral ischemia. Stroke 32, 1028–1035. 10.1161/01.STR.32.4.1028 11283407

[B100] Zang J. Wu Y. Su X. Zhang T. Tang X. Ma D.. (2020). Inhibition of PDE1-B by vinpocetine regulates microglial exosomes and polarization through enhancing autophagic flux for neuroprotection against ischemic stroke. Front. Cell Dev. Biol. 8:616590. 10.3389/fcell.2020.616590 33614626 PMC7889976

[B101] Zhou Y. Zhou B. Pache L. Chang M. Khodabakhshi A. H. Tanaseichuk O.. (2019). Metascape provides a biologist-oriented resource for the analysis of systems-level datasets. Nat. Commun. 10:1523. 10.1038/s41467-019-09234-6 30944313 PMC6447622

