# Supplementary material for: Novel potential neuroprotective targets for DengZhanXiXin injection in middle cerebral artery occlusion rats recommended by quantitative proteomics and simulated docking
Source: Front Neurosci. 2025 Jul 7;19:1499214. doi: 10.3389/fnins.2025.1499214 (PMC12277316; doi:10.3389/fnins.2025.1499214)
Supplement: Supplementary file 2 [file Table_2.DOCX]

**Supplementary tables**

Supplementary table S2. The Molecular function and biological process category of 39 differentially expressed proteins regulated by 7d-treatment of DZXI in the infarcted hemispheres of MCAO rats.

| NO. | Protein name | Molecular function | Biological process |
| --- | --- | --- | --- |
| **Anti-inflammatory proteins** | | | |
| 1 | Annexin A1 | Calcium-dependent phospholipid binding, double-stranded DNA helicase activity, single-stranded DNA binding, phospholipase A2 inhibitor activity | Actin cytoskeleton reorganization, adaptive immune response, innate immune response, positive regulation of wound healing, cellular response to hydrogen peroxide, gliogenesis |
| 2 | Annexin A2 | Actin filament binding, calcium channel activity, calcium ion binding, calcium-dependent phospholipid binding, serine-type endopeptidase inhibitor activity | Angiogenesis, fibrinolysis, positive regulation of low-density lipoprotein particle clearance, positive regulation of plasminogen activation, regulation of neurogenesis |
| 3 | Annexin | Calcium-dependent phospholipid binding, calcium ion binding | Negative regulation of coagulation |
| **Calcium-dependent phospholipid-binding proteins** | | | |
| 4 | Copine 4 | Calcium-dependent phospholipid binding | Cellular response to calcium ion |
| 5 | Copine 6 | Calcium-dependent phospholipid binding, phosphatidylserine binding | Cellular response to calcium ion, positive regulation of dendrite extension |
| 6 | Copine-7 | Calcium-dependent phospholipid binding, metal ion binding | Cellular response to calcium ion |
| **Integral synaptic vesicle membrane protein** | | | |
| 7 | Synaptotagmin II | Calcium-dependent phospholipid binding, calcium ion binding | Neurotransmitter secretion, positive regulation of dendrite extension |
| **Detoxification enzymes (ROS and xenobiotics metabolism)** | | | |
| 8 | Glutathione S-transferase | Glutathione transferase activity, identical protein binding | Glutathione metabolic process, xenobiotic metabolic process |
| 9 | Retinal dehydrogenase 2 | 3-chloroallyl aldehyde dehydrogenase activity, aldehyde dehydrogenase (NAD+) activity, retinal dehydrogenase activity | 9-cis-retinoic acid biosynthetic process, blood vessel development, positive regulation of apoptotic process, regulation of vascular endothelial cell proliferation, retinoic acid metabolic process |
| 10 | Aldo-keto reductase family 1 member B10 | Alcohol dehydrogenase (NADP+) activity, geranylgeranyl reductase activity, indanol dehydrogenase activity, NADP-retinol dehydrogenase activity | Cellular detoxification of aldehyde, daunorubicin metabolic process, farnesol catabolic process, retinol metabolic process |
| **Structural constituents of myelin sheath** | | | |
| 11 | 2-hydroxyacylsphingosine 1-beta-galactosyltransferase | 2-hydroxyacylsphingosine 1-beta-galactosyltransferase activity, N-acylsphingosine galactosyltransferase activity, UDP-galactose:glucosylceramide beta-1,4-galactosyltransferase activity | Cytoskeleton organization, galactosylceramide biosynthetic process, glycosphingolipid biosynthetic process, myelination, paranodal junction assembly |
| 12 | Myelin basic protein | Structural constituent of myelin sheath | MAPK cascade, immune response, chemical synaptic transmission, axon ensheathment |
| 13 | Myelin proteolipid protein | Protein-containing complex binding, structural constituent of myelin sheath | AMPA selective glutamate receptor signaling pathway, inflammatory response, myelination, positive regulation of calcium ion transmembrane transport, positive regulation of cell migration |
| **Neurofilament structural components** | | | |
| 14 | Neurofilament light polypeptide | Structural constituent of cytoskeleton, structural constituent of postsynaptic intermediate filament cytoskeleton, phospholipase binding, protein C-terminus binding, protein-macromolecule adaptor activity | Anterograde axonal transport, retrograde axonal transport, axonal transport of mitochondrion, positive regulation of axonogenesis, intermediate filament polymerization or depolymerization, negative regulation of motor neuron apoptotic process, neuromuscular process controlling balance |
| 15 | Neurofilament medium polypeptide | Structural constituent of cytoskeleton, protein-containing complex binding, signaling receptor binding, toxic substance binding | Axo-dendritic transport, axon regeneration, cellular response to oxidative stress, intermediate filament polymerization or depolymerization, regulation of axon diameter |
| 16 | Neurofilament heavy polypeptide | Structural constituent of cytoskeleton, microtubule binding, protein-macromolecule adaptor activity | Axon development |
| **Carbonic anhydrases** | | | |
| 17 | Carbonic anhydrase 1 | Arylesterase activity, carbonate dehydratase activity, hydro-lyase activity, zinc ion binding | One-carbon metabolic process |
| 18 | Carbonic anhydrase 2 | Arylesterase activity, carbonate dehydratase activity, hydro-lyase activity, zinc ion binding | Angiotensin-activated signaling pathway, cellular response to fluid shear stress, carbon dioxide transport, one-carbon metabolic process, neuron cellular homeostasis, positive regulation of cellular pH reduction, positive regulation of synaptic GABAergic transmission |
| 19 | Carbonic anhydrase 3 | Carbonate dehydratase activity, hydro-lyase activity, phosphatase activity, zinc ion binding, nickel cation binding | One-carbon metabolic process, response to oxidative stress |
| **Glutamate receptors** | | | |
| 20 | Glutamate receptor 1 | Ionotropic glutamate receptor activity | Signaling, transmembrane transport |
| 21 | Metabotropic glutamate receptor 2 | Adenylate cyclase inhibiting G protein-coupled glutamate receptor activity, calcium channel regulator activity, scaffold protein binding | Adenylate cyclase-inhibiting G protein-coupled glutamate receptor signaling pathway, glutamate homeostasis, regulation of glutamate secretion, regulation of dopamine secretion, regulation of protein kinase B signaling |
| 22 | Protein cornichon homolog 2 | Transporter activity, molecular transducer activity | Negative regulation of anterograde synaptic vesicle transport, negative regulation of receptor localization to synapse, regulation of AMPA receptor activity, regulation of NMDA receptor activity, regulation of membrane potential |
| 23 | Shisa family member 6 | Ionotropic glutamate receptor binding | Excitatory chemical synaptic transmission, regulation of AMPA receptor activity, postsynaptic neurotransmitter receptor diffusion trapping, negative regulation of canonical Wnt signaling pathway |
| **γ-aminobutyric acid (GABA) receptors** | | | |
| 24 | Gamma-aminobutyric acid receptor subunit gamma-2 | GABA-gated chloride ion channel activity, neurotransmitter receptor activity, chloride channel activity | Gamma-aminobutyric acid signaling pathway, chloride transmembrane transport, regulation of postsynaptic membrane potential, cellular response to histamine |
| 25 | Gamma-aminobutyric acid receptor subunit beta-1 | GABA-gated chloride ion channel activity, neurotransmitter receptor activity, anion channel activity | Gamma-aminobutyric acid signaling pathway, chloride transmembrane transport, regulation of membrane potential, cellular response to histamine, central nervous system neuron development |
| **Actin-binding proteins** | | | |
| 26 | Erythrocyte membrane protein band 4.1-like 3 | Actin binding, structural molecule activity | Actomyosin structure organization, cortical actin cytoskeleton organization, myelin maintenance, paranodal junction maintenance, protein localization to plasma membrane, regulation of cell growth |
| 27 | Transgelin | Actin filament binding | Epithelial cell differentiation, muscle organ development |
| 28 | Myl9 protein | Calcium ion binding, myosin heavy chain binding | Myofibril assembly |
| **Energy metabolism** | | | |
| 29 | Cytochrome c oxidase subunit 2 | Cytochrome-c oxidase activity | Generation of precursor metabolites and energy, transmembrane transport |
| **Acute-phase proteins** | | | |
| 30 | Alpha-1-acid glycoprotein | Protein binding, small molecule binding | Acute-phase response, regulation of immune system process |
| 31 | Sestrin 3 | Leucine binding, oxidoreductase activity acting on peroxide as acceptor | Cellular response to glucose starvation, cellular response to leucine starvation, negative regulation of TORC1 signaling, positive regulation of macroautophagy, response to insulin, regulation of response to reactive oxygen species |
| **Cell signaling** | | | |
| 32 | Calcium/calmodulin-dependent 3',5'-cyclic nucleotide phosphodiesterase 1B | 3',5'-cyclic-AMP phosphodiesterase activity, 3',5'-cyclic-GMP phosphodiesterase activity, calmodulin binding, metal ion binding | Cellular response to macrophage colony-stimulating factor stimulus, monocyte differentiation, regulation of dopamine metabolic process, serotonin metabolic process, regulation of neurotransmitter levels |
| 33 | Calcium/calmodulin-dependent 3',5'-cyclic nucleotide phosphodiesterase 1C | 3',5'-cyclic-AMP phosphodiesterase activity, 3',5'-cyclic-GMP phosphodiesterase activity, calmodulin binding, metal ion binding | Negative regulation of insulin secretion involved in cellular response to glucose stimulus, response to calcium ion, signal transduction |
| 34 | cAMP and cAMP-inhibited cGMP 3',5'-cyclic phosphodiesterase 10A | 3',5'-cyclic-AMP phosphodiesterase activity, 3',5'-cyclic-GMP phosphodiesterase activity, metal ion binding | cAMP catabolic process, cGMP catabolic process, negative regulation of cAMP-mediated signaling, negative regulation of cGMP-mediated signaling, regulation of protein kinase A signaling |
| 35 | Protein kinase C gamma type | ATP binding, calcium-dependent protein kinase C activity, protein serine/threonine/tyrosine kinase activity, zinc ion binding | Presynaptic modulation of chemical synaptic transmission, intracellular signal transduction, negative regulation of protein catabolic process, negative regulation of neuron apoptotic process, protein autophosphorylation |
| 36 | Protein kinase AMP-activated non-catalytic subunit gamma 2 | AMP, ADP and ATP binding, cAMP-dependent protein kinase regulator activity, phosphorylase kinase regulator activity | Cellular response to glucose starvation, glycogen metabolic process, negative regulation of protein kinase activity, protein phosphorylation, intracellular signal transduction, regulation of fatty acid metabolic process, regulation of glycolytic process |
| 37 | Diacylglycerol kinase gamma | ATP binding, calcium ion binding, lipid binding, diacylglycerol kinase activity | Diacylglycerol metabolic process, glycerolipid metabolic process, lipid phosphorylation, phosphatidic acid biosynthetic process, intracellular signal transduction, negative regulation of protein kinase C signaling, regulation of dendrite development |
| 38 | Adenylate cyclase 5 | Adenylate cyclase activity, ATP binding, metal ion binding | Adenylate cyclase-dependent dopamine receptor signaling pathway, cAMP biosynthetic process, intracellular signal transduction, positive regulation of cytosolic calcium ion concentration, locomotory behavior |
| 39 | Guanine nucleotide-binding protein G(olf) subunit alpha | G protein-coupled receptor binding, GTP binding, GTPase activity | Activation of adenylate cyclase activity, adenylate cyclase-activating G protein-coupled receptor signaling pathway, cellular response to dopamine, regulation of long-term synaptic depression |

Supplementary table S3. Molecular binding affinities from virtual docking analysis (kcal/mol) between five compounds of DZXI and 8 proteins respectively.

|  | scutellarin | 3,4-O-dicaffeoylquinic acid | 3,5-O-dicaffeoylquinic acid | 4,5-O-dicaffeoylquinic acid | erigoster B |
| --- | --- | --- | --- | --- | --- |
| PDE10A | -6.31 | -7.96 | -6.98 | -7.13 | -6.86 |
| PRKCG | -6.17 | -7.14 | -7.37 | -7.07 | -6.95 |
| DGKG | -6.09 | -6.83 | -6.77 | -6.57 | -6.71 |
| GNAL | -6.01 | -6.85 | -6.63 | -6.75 | -6.78 |
| GRIA1 | -6.79 | -7.12 | -6.47 | -7.32 | -7.06 |
| GRM2 | -6.07 | -7.05 | -6.17 | -6.49 | -6.69 |
| MT-CO2 | -6.75 | -7.19 | -6.71 | -7.38 | -6.76 |
| CAR3 | -6.03 | -6.94 | -6.91 | -7.40 | -6.84 |
